# Electric‐Field Catalysis on Carbon Nanotubes in Electromicrofluidic Reactors: Monoterpene Cyclizations

**DOI:** 10.1002/anie.202417333

**Published:** 2024-11-14

**Authors:** Augustina Jozeliūnaitė, Shen‐Yi Guo, Naomi Sakai, Stefan Matile

**Affiliations:** ^1^ Department of Organic Chemistry University of Geneva Geneva Switzerland; ^2^ National Centre of Competence in Research (NCCR) Molecular Systems Engineering BPR 1095 Basel Switzerland

**Keywords:** Oriented external electric fields, anion-π catalysis, cation-π catalysis, microfluidics, flow chemistry, carbon nanotubes, terpenes, cyclizations, hydride shifts

## Abstract

The control over the movement of electrons during chemical reactions with oriented external electric fields (OEEFs) has been predicted to offer a general approach to catalysis. Recently, we suggested that many problems to realize electric‐field catalysis in practice under scalable bulk conditions could possibly be solved on multiwalled carbon nanotubes in electromicrofluidic reactors. Here, we selected monoterpene cyclizations to assess the scope of our system in organic synthesis. We report that electric‐field catalysis can function by stabilizing both anionic and cationic transition states, depending on the orientation of the applied field. Moreover, electric‐field catalysis can promote reactions which are barely accessible by general Brønsted and Lewis acids and field‐free anion‐π and cation‐π interactions, and drive chemoselectivity toward intrinsically disfavored products without the need for pyrene interfacers attached to the substrate to prolong binding to the carbon nanotubes. Finally, interfacing with chiral organocatalysts is explored and evidence against contributions from redox chemistry is provided.

## Graphical abstract

Studies on the cyclization of monoterpenes on carbon nanotubes in electromicrofluidic devices support that electric‐field catalysis under scalable bulk conditions can stabilize both anionic and cationic transition states, can provide access to reactions general Brønsted acid, Lewis acid, anion‐π and cation‐π catalysts do not to really catalyze, and drive chemoselectivity toward intrinsically disfavored products.

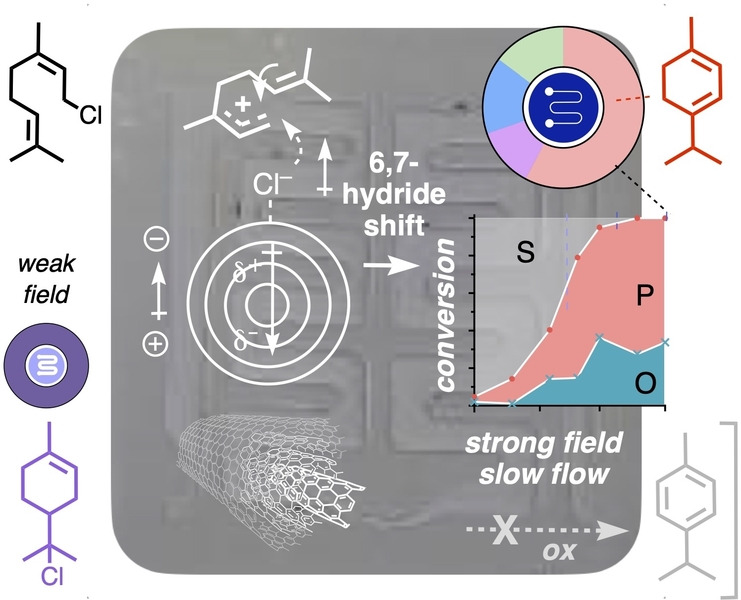

In chemical reactions, electrons move from one place to another within the same or a different molecule. Theory[^1^, ^2^, ^3^, ^4^, ^5^, ^6^, ^7^, ^8^, ^9^] and enzymology[^10^, ^11^, ^12^, ^13^, ^14^, ^15^] predict that if we could accelerate and direct this charge translocation with oriented external electric fields (OEEFs), we could fundamentally change organic synthesis. However, progress with electric‐field catalysis has been hampered by an overwhelming accumulation of practical problems under scalable bulk conditions. Despite pioneering efforts to address these challenges, experimental support for electric‐field catalysis has been mostly limited to more exotic conditions such as single molecules on STM tips, water droplets or intriguing self‐built devices.[^1^, ^2^, ^3^, ^4^, ^16^, ^17^, ^18^, ^19^, ^20^, ^21^, ^22^, ^23^, ^24^, ^25^, ^26^, ^27^, ^28^]

Last year, we realized that electromicrofluidic reactors with multiwalled carbon nanotube (MWCNT)[^29^, ^30^, ^31^, ^32^, ^33^, ^34^] coated electrodes could possibly overcome these intrinsic challenges with electric‐field catalysis in combination with anion‐π catalysis,[^34^, ^35^, ^36^, ^37^, ^38^, ^39^] lifting both principles to a new level of significance.^[40]^ Electromicrofluidic reactors have been constructed for electroorganic redox chemistry under scalable flow conditions.[^41^, ^42^, ^43^, ^44^, ^45^] From the point of view of electric‐field catalysis, their characteristics seemed well suited to also enable supramolecular systems catalysis without electron transfer. They are composed of two 5×5 cm electrodes, usually graphite and Pt, that sandwich a 250 μm thin foil with the flow channel (Figures 1, S1). The resulting short distance between the electrodes is critical to access sufficient conductivity without the need of electrolytes, which inhibit ion‐π interactions, and to establish favorable local catalyst to substrate ratios. Drop‐casting of MWCNTs onto the graphite electrode was essential to increase the effective surface area and conductivity, maximize local electric fields and, most importantly, merge electric‐field catalysis with an/cat‐ion‐π catalysis (Figure 1).

**Figure 1:**
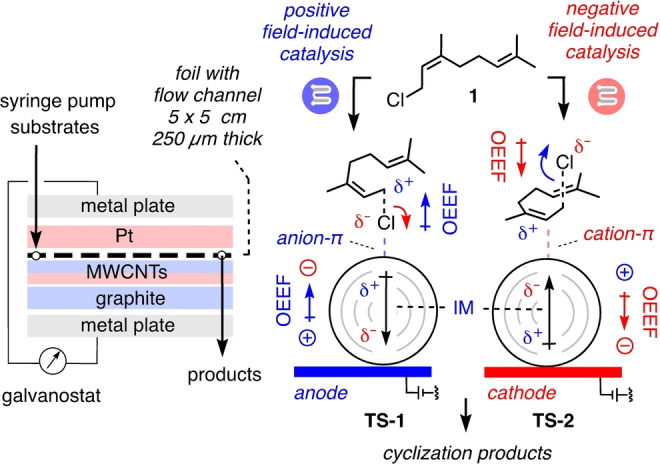
The electromicrofluidic system devised for electric‐field catalysis of terpene cyclization, with substrate **1** and notional transition states of the first step, a C−Cl bond cleavage. A 250 μm thin foil with the flow channel is sandwiched between MWCNT‐coated graphite and Pt electrodes. The graphite electrode used as anode (blue) or cathode (red) produces OEEFs of opposite direction (colored tagged arrows pointing from positive to negative). To activate terpene cyclization, these OEEFs should i) directly catalyze C−Cl bond cleavage (curved arrows, movement of electrons) and ii) polarize MWCNTs with induced macrodipoles (IM, black tagged arrows, positive to negative) to induce anion‐/cation‐π interactions for catalysis of the same C−Cl bond cleavage.

Proof‐of‐principle for electric‐field catalysis on MWCNTs in electromicrofluidic reactors has been secured with non‐selective single epoxide opening ether formation as a model reaction.^[40]^ After this breakthrough, the next question was whether this approach would fulfill the promise of more general access to scalable electric‐field catalysis in organic synthesis. To address this question, monoterpene cyclizations were selected as mechanistically rich and revealing processes with large product space.[^46^, ^47^, ^48^, ^49^, ^50^, ^51^, ^52^, ^53^, ^54^, ^55^, ^56^, ^57^, ^58^, ^59^, ^60^, ^61^, ^62^, ^63^, ^64^, ^65^, ^66^, ^67^] In nature, the richness of monoterpene natural products, most popular as flavors and fragrances, emerges from the cyclization of GPP.[^68^, ^69^, ^70^] With the similarly activated monoterpene neryl chloride **1** as substrate, activation on MWCNTs in electromicrofluidic devices was conceivable with positive (blue) and negative electric fields (red, Figure 1). Namely, the polarization of MWCNTs by electric fields will induce large macrodipoles. These macrodipoles will increase the π acidity or π basicity on the nanotube surface, depending on the direction of the applied electric field. This induced π acidity and π basicity will enable strong anion‐π and cation‐π interactions, respectively, to support the electric fields which catalyze the reaction of interest. In **TS‐1**, this is the cleavage of the C−Cl bond of neryl chloride **1**. The direct electric‐field catalysis of this bond cleavage by the applied OEEF will be supported by anion‐π interactions between the released chloride and the locally π‐acidic polarized MWCNTs. In **TS‐2**, the same C−Cl bond is cleaved by OEEFs in opposite direction, supported by cation‐π interactions between the allylic carbocation intermediate and the locally π‐basic MWCNTs.

Reversible release of the anionic leaving group in substrate **1** affords the allylic carbocation intermediate **RI‐1** (Figure 2a). After cyclization, the terpinyl carbocation **RI‐2** can proceed with charge recombination into α‐terpinyl chloride **2**, deprotonation to afford terpinolene **3** and limonene **4**, or another cyclization toward bicyclic monoterpenes like the prickly cooling camphor, borneol, pinene, and so on. Alternatively, a 6,7‐hydride shift can transform **RI‐2** into **RI‐3**, which then can deprotonate to yield γ‐terpinine **5** and α‐terpinine **6** in addition to **3**, or continue to cyclize into more complex monoterpenes like the spicy sabinene or α‐tujone, the legendary active compound in absinth.^[71]^ From the terpinenes **5** and **6**, (auto)oxidation finally can lead to the aromatic *p*‐cymene **7**.[^49^, ^72^]

**Figure 2:**
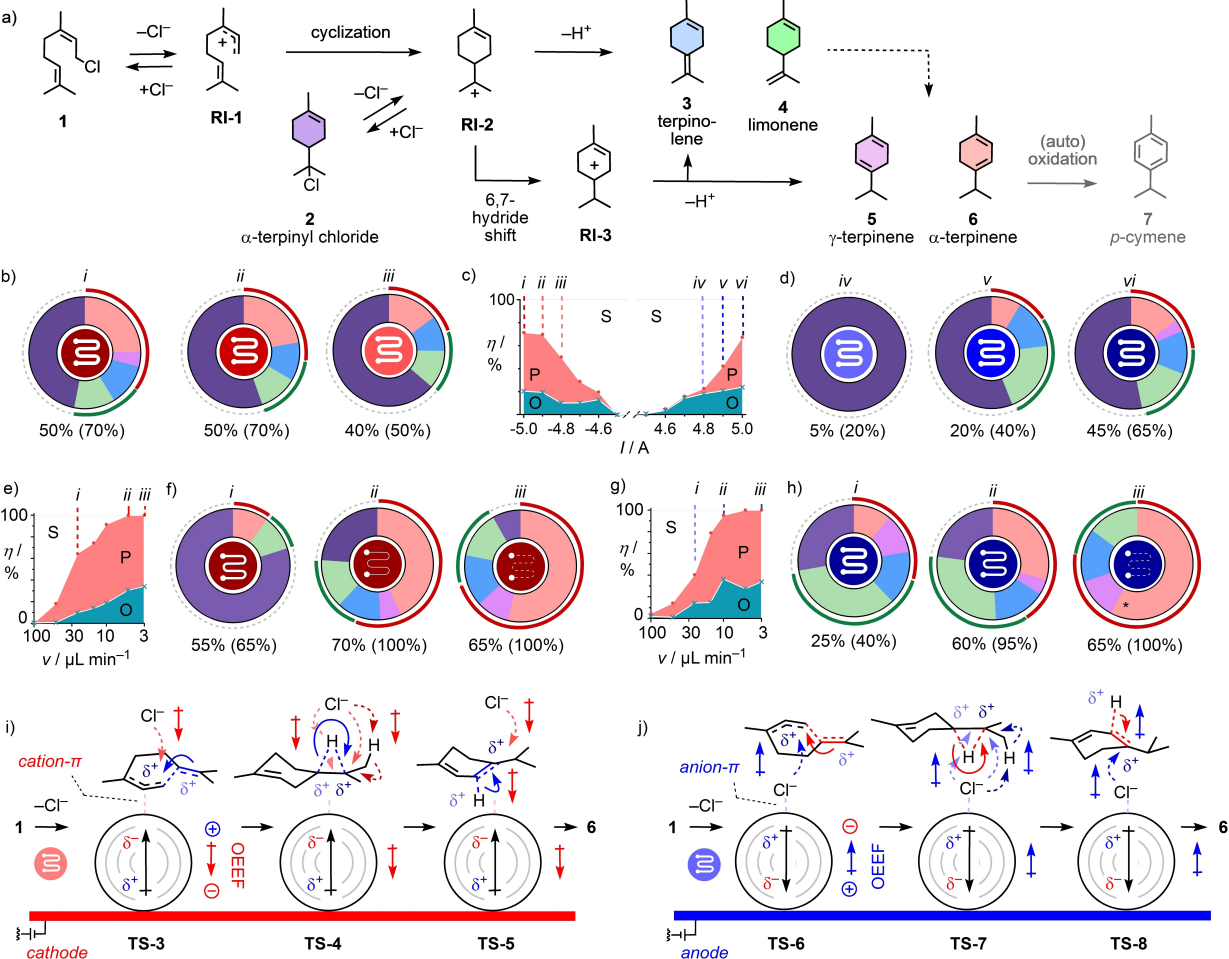
Cyclization of **1** (100 mM) in toluene catalyzed by negative (b/c, e/f) and positive (c/d, g/h) electric fields, with cyclization mechanisms (a, i, j) and color‐coded products **2**–**6**. (c, e, g) Substrate (S), identified products (P) and unidentified others (O) after passing once through the electromicrofluidic reactor as a function of the applied current (c, *v*=15 μL min^−1^) and the flow rate (e, g, *I*=±5.0 A). Symbols inside doughnut charts indicate orientation (red, MWCNT‐coated graphite as cathode, referred to as negative electric fields; blue, MWCNTs as anode, positive electric fields) and strength (light/dark color) of the applied field, and flow velocity (fast=thick solid, slow=thin dashed curly lines; peripheral circular arcs distinguish mechanisms with deprotonation and with (red) and without (green) hydride shift; *including small amounts of *p*‐cymene **7** (7 %, Figure S32). (i, j) Speculative transition states **TS‐3** to **TS‐8** stabilized by negative (i, red tagged arrows) and positive electric fields (j, blue tagged arrows). Electron translocation during reactions is indicated by curved arrows, opposite color and directions indicate activation, identical color and orientation inactivation by electric fields. **TS‐4** and **TS‐7** speculate on chemoselectivity, emphasizing how alignment of the carbocation‐chloride ion pairs to increasing electric fields could activate the hydride shifts to reach **5** and **6** (curved arrows, color opposite to field) and hinder chloride addition to **2** (dashed curved arrows, same light color) and proton elimination to **3** and **4** (dashed curved arrows, same dark color).

In the absence of OEEFs, 100 mM neryl chloride **1** (Scheme S1, Figure S37) in toluene did not react during one passage through the electromicrofluidic reactor (Figure 2c). This result was of central importance because it demonstrated that without electric field, neither anion‐π nor cation‐π interactions on MWCNTs can catalyze terpene cyclization. Negative electric‐field catalysis was only detectable below *I*=−4.5 A[^73^, ^74^, ^75^] (Figures 2c, S29). According to gas chromatography (GC) analyses compared to internal standards (Figures S12–S27, Table S1),^[53]^ total conversion (P+O) after one passage through the microfluidic reactor increased with the external field up to *η*=70 % at *I*=−5.0 A. Most main products were identified and quantified by comparing their GC traces with those of commercial samples, and their sum afforded yields P (Figures 2c, red; S3, S29, Table S2). Small peaks and missing matter, lost by evaporation, etc, were summarized as others (O, Figure 2c, teal). At weaker fields, most of the identified products was α‐terpinyl chloride **2**, followed by deprotonation products **3** and **4** (Figure 2b, *iii*, purple, blue, green). Stronger fields gave more hydride‐shifted terpinenes **5** (pink) and particularly **6** (red) at the cost of **2**, dropping below 50 % (Figure 2b, *i*, purple).

Negative electric‐field catalysis increased with slower flow up to completion within one passage through the reactor (Figures 2e, S4, S31, Table S4). With decelerated flow, product distribution shifted from the **RI‐2** equivalent α‐terpinyl chloride **2** (Figure 2f, *i*, purple) to the hydride‐shifted α‐terpinine **6** (Figure 2f, *iii*, red).

With positive electric‐field catalysis, stronger fields were needed to turn on cyclization (Figures 2c, S3, S30, Table S3). However, saturation‐free increase from there led to comparable total substrate consumption (P+O) and assigned product yield (P) at *I*=+5.0 A[^73^, ^74^, ^75^] (Figure 2c). Similar trends were observed upon decelerating flow (Figure 2g, S4, S32, Table S5). Chemoselectivity also shifted to terpinines **5** and **6** (Figure 2h, *iii*, red+pink). Overall more responsive than with negative electric fields, positive electric‐field catalysis started with only (Figure 2d, *iv*) and ended without any α‐terpinyl chloride **2**, and with more terpinines **5** (pink) and **6** (red) at strongest field and slowest flow (Figure 2h, *iii*, purple).

These results indicated that initial C−Cl bond cleavage can be promoted in both orientations of the field (**TS‐1**, **TS‐2**, Figure 1). From the allylic carbocation **RI‐1**, electric fields appeared to primarily extend the lifetime of the carbocation[^46^, ^47^, ^48^] by aligning carbocation‐chloride ion pairs in **TS‐3** and **TS‐6**, thus preventing charge recombination back to **1** independent of their orientation (Figure 2i, j, dashed curved arrows), while supporting cyclization into **RI‐2** (Figure 2i, j, solid curved arrows).

After cyclization into **RI‐2**, remote control by electric fields over charge recombination (**2**), deprotonation (**3**, **4**) and hydride shift plus deprotonation (**3**, **5**, **6**) could account for the emergence of chemoselectivity. The sharp increase of the product ratio **6**/**2** at the strongest field and slowest flow despite nearly full conversion supported that **2** can be returned to **RI‐2** by renewed charge separation catalyzed by electric fields. Treated under the same conditions, limonene **4** remained intact, indicating that the electric field promoted the 6,7‐hydride shift rather than the isomerization of products **3** or **4** (Figure 2a). Beside hindering chloride addition with field‐oriented carbocation‐chloride ion pairs, field‐induced chemoselectivity thus could possibly originate from the best stabilization of the most polarizable delocalized carbocation during hydride shifts in **TS‐4** and **TS‐7** with electric fields, an/cation‐π interactions and oriented ion pairing, affording, after deprotonation through **TS‐5** and **TS‐8**, terpinines **5** and mostly **6** (Figure 2f, h, *iii*; 2i, j, solid vs dashed curved arrows for promoted vs hindered electron movements by field, respectively). Although entirely speculative at this point, these mechanisms are in agreement with existing computational data with specific relevance for the topic beyond the general concept of electric‐field catalysis.[^31^, ^66^, ^76^]

While several elegant approaches to cyclize monoterpenes into interesting product mixtures have been reported,[^46^, ^47^, ^48^, ^49^] terpene cyclization with general Brønsted and Lewis acids usually gives hydride‐shifted products **5**/**6** at only poor yields through isomerization of **3**/**4**.[^49^, ^50^, ^51^, ^52^, ^53^] The emergence and evolution of chemoselectivity from **2** to **3**/**4** and then the hydride‐shifted **5**/**6** with increasing field and decreasing flow (Figure 2f, h, *iii*) thus supported that electric‐field catalysis on carbon nanotubes in electromicrofluidic reactors can provide access to reactions general Brønsted and Lewis acid and an/cation‐π catalysts do not catalyze well, and drive chemoselectivity toward intrinsically disfavored products.

We further explored the effect of electric fields on organocatalysts at the surface of MWCNTs using chiral catalyst **8** (Figure 3). This Jacobsen catalyst has previously been shown to promote the same terpene cyclization reaction assisted by the activated urea for chloride binding, and the aromatic surface for stabilization of carbocation intermediates.^[48]^ Based on the well exploited efficient π stacking of PAHs, like pyrene and also phenanthrene derivatives, on carbon nanotubes for catalysis,[^29^, ^33^, ^40^, ^77^, ^78^, ^79^] we hypothesized that the same aromatic surface in catalyst **8** could serve as an interfacer to position and orient the catalyst‐substrate complex on MWCNTs. Catalyst **8** was easily synthesized following the reported procedures (Scheme S2).^[48]^

**Figure 3:**
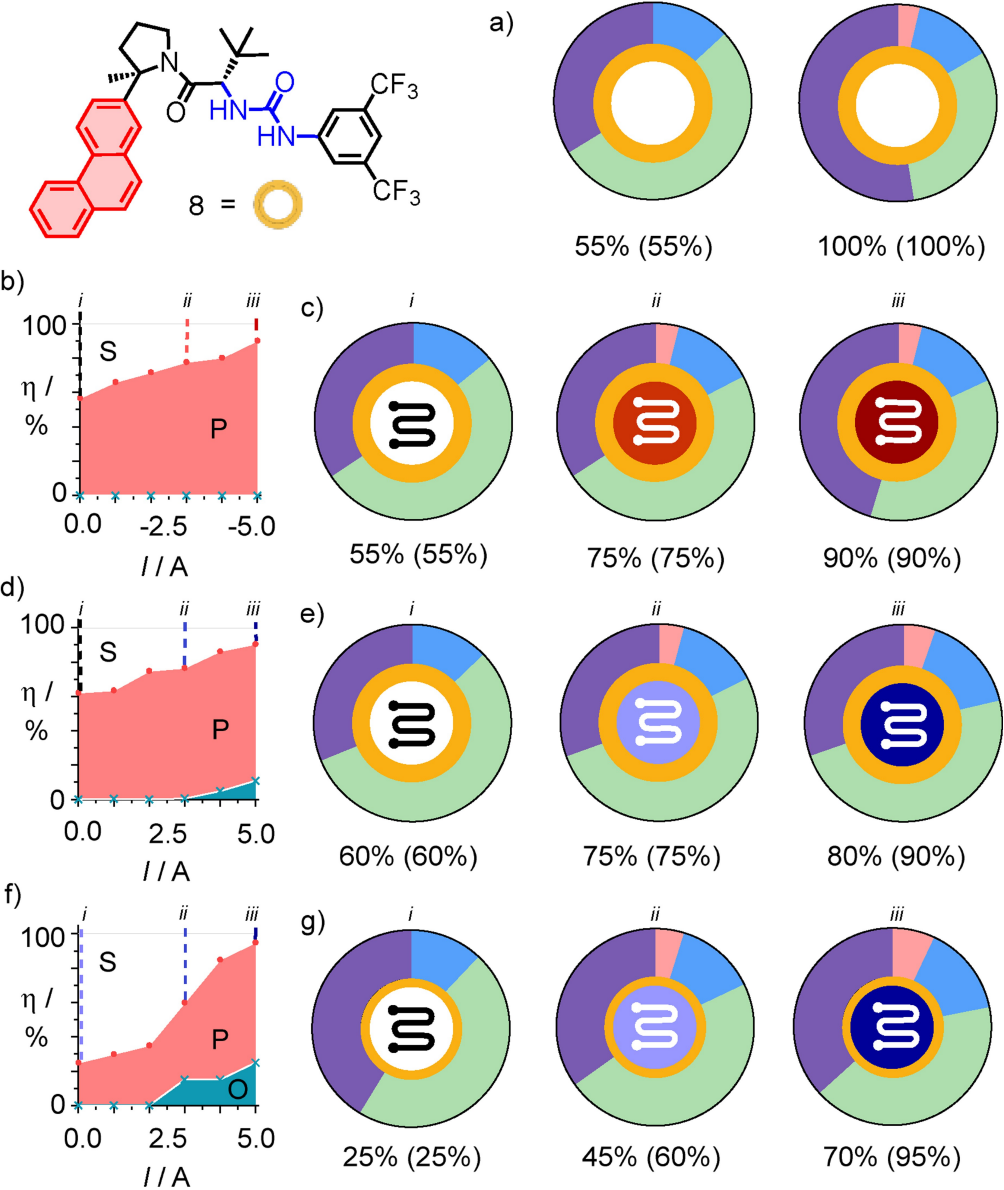
Interfacing of the Jacobsen catalyst **8** (gold, a–e, 100 mol %; f, g, 30 mol %) with negative (b, c) and positive electric‐field catalysis (d–g) to cyclize 100 mM **1** in toluene. (a) Product distribution for partial (30 min) and full conversion of **1** (22 h) in electromicrofluidics‐free solution. (b, d, f) Substrate (S), product (P) and others (O) after one passage through the electromicrofluidic reactor as a function of the applied current (*v*=10 μL min^−1^), and (c, e, g) doughnut charts for product distributions, encoded as in Figure 2.

Consistent with the precedence,^[48]^ **1** cyclized in bulk solution in the presence of 100 mol % **8** into mainly limonene **4**, but also much charge‐recombined **2** and very little hydride‐shifted **6** (Figures 3a, S5, S33, Table S6). Injected simultaneously from separate syringes through a T‐mixer into the electromicrofluidic reactor, 100 mol % catalyst **8** caused the conversion of around 60 % substrate **1** already without electric field (Figure 3b, d). Gradually increasing conversion toward 90 % at *I*=±5.0 A was nearly independent on the direction of the applied field (Figure 3b, d) and without significant changes in chemoselectivity (Figures 3c, e, S6, S7, Table S7). Access to detectable rate enhancements also at weaker fields was interesting and meaningful because catalyst **8** lowered the activation energy enough to operate even without field. Without catalyst **8**, monoterpene cyclization did not occur without electric fields in the electromicrofluidic reactor, and much stronger fields were therefore needed to turn on the reaction (Figure 2).

With only 30 mol % **8**, one passage through the device without field gave less conversion (Figures 3f, S7, Table S7). Positive electric‐field catalysis could thus more significantly increase conversion and, at strongest field, roughly double also the small percentage of hydride‐shifted **6** (Figures 3f, g). Decelerated flow (analogous to Figure 2e, g, Figure S8, Table S8), temperature variations (Figure S9, Table S9) and the presence of base (lutidine, 2,6 di‐*t‐*Bu pyridine, Figure S10, Tables S10, S11)^[48]^ failed to significantly change chemoselectivity (Figures S8–S10). In perfect agreement with the original results with catalyst **8** in solution,^[48]^ chiral products of substrate **1** were also obtained in racemic form with electric‐field catalysis (not shown). Overall, with regard to conversion enhancements and chemoselectivity for disfavored products, the effect of positive and negative electric fields was obfuscated rather than amplified by the Jacobsen catalyst **8**.

All our experiments were conducted under constant current conditions, under which, voltages can vary to a small extent depending on nanotube materials, electrode preparation, age, etc (Tables S2–S11). Moreover, the measured macroscopic voltages most likely differed fundamentally from the effective local electric fields accounting for catalysis on the polarized MWCNTs.[^73^, ^74^, ^75^] To illustrate this essential characteristic and probe for possible contributions from electron transfer and redox chemistry, the oxidation of hydroquinone **9** to quinone **10** was selected (Figures 4, S11).^[80]^ This oxidation would be easily detectable as the replacement of the hydroquinone absorption at around 300 nm by an intense quinone absorption at around 240 nm (Figure 4a). The reported oxidation potential is at *E*=400 mV vs SCE.^[80]^ However, after passing hydroquinone **9** through the electromicrofluidic reactor under standard conditions, only traces of quinone **10** were obtained with currents up to *I*=+5.0 A and voltages up to *V*=1.36 V (Figure 4b). This result was in agreement with negligible aromatization of **5**/**6** into *p*‐cymene **7**, which can be nearly spontaneous without extra oxidants in other supramolecular functional systems[^49^, ^72^] but was observed here in trace amounts mostly at extreme positive field and slow flow (0–8 %), confirming negligible contributions from redox chemistry under these conditions (Figures 2, S29–S32, Tables S4, S5). The drop‐casted MWCNTs, however, were essential to generate significant currents. Different mechanisms deserve consideration, including field‐induced nanotube reorientation, transient electron transfer and tunneling, with obvious opportunities to achieve strong local electric fields for efficient catalysis.[^73^, ^74^, ^75^]

**Figure 4:**
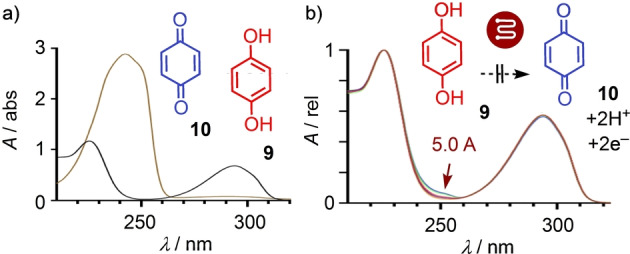
Assessment of electron transfer under experimental conditions. a) Absorption spectra of hydroquinone **9** (black) and equimolar **10** (brown, oversaturated at A≫1.5). b) Absorption spectra of reaction mixture after passing once through the electromicrofluidic reactor at increasing current up to *I*=±5.0 A (blue, apparent voltage *V*=1.36 V).

Taken together, the objective of this study was to assess whether our approach using eletromicrofluidics on carbon nanotubes can meet the high expectations for electric‐field catalysis in organic synthesis under scalable bulk conditions. We find that, for terpene cyclization, pyrene interfacers attached to the substrate^[40]^ are not needed and that, less surprising but worth confirming, both anionic and cationic transition states can be stabilized. Most importantly, electric‐field catalysis provides access to reactions conventional Brønsted and Lewis acid struggle to catalyze. Under identical conditions, field‐free anion‐π and cation‐π catalysis also fail to catalyze terpene cyclizations. Of similarly critical importance is evidence that changing fields can change chemoselectivity of a reaction and selectively accelerate the formation of intrinsically disfavored products. These results support that electric‐field catalysis on carbon nanotubes in electromicrofluidic devices could become generally useful in organic synthesis. However, much more data on diverse reactions will be needed to formulate general recommendations for use in practice. Preliminary results in this direction exist, including constructive interfacing with organocatalysts, intermolecular reactions and asymmetric catalysis.

## Conflict of Interests

The authors declare no conflict of interest.

## Supporting information

As a service to our authors and readers, this journal provides supporting information supplied by the authors. Such materials are peer reviewed and may be re‐organized for online delivery, but are not copy‐edited or typeset. Technical support issues arising from supporting information (other than missing files) should be addressed to the authors.

Supporting Information

## Data Availability

The data that support the findings of this study are openly available in zenodo at https://doi.org/10.5281/zenodo.13165225.
