# Supplementary material for: Electric‐Field Catalysis on Carbon Nanotubes in Electromicrofluidic Reactors: Monoterpene Cyclizations
Source: Angew Chem Int Ed Engl. 2024 Nov 14;64(4):e202417333. doi: 10.1002/anie.202417333 (PMC11753599; doi:10.1002/anie.202417333)
Supplement: Supplementary file 1 — Supporting Information [file ANIE-64-e202417333-s001.pdf]

Angewandte Chemie
Eine Zeitschrift der Gesellschaft Deutscher Chemiker

Supporting Information

Electric-Field Catalysis on Carbon Nanotubes in Electromicrofluidic Reactors: Monoterpene Cyclizations

*A. Jozeliūnaitė, S.-Y. Guo, N. Sakai, S. Matile**

Supporting Information

Electric-Field Catalysis on Carbon Nanotubes in Electromicrofluidic Reactors: Monoterpene Cyclizations

Augustina Jozeliūnaitė, Shen-Yi Guo, Naomi Sakai, and Stefan Matile*[a,b]

[a]Department of Organic Chemistry, University of Geneva, Geneva, Switzerland

[b]National Centre of Competence in Research (NCCR) Molecular Systems Engineering, BPR 1095, Basel, Switzerland

*E-mail: stefan.matile@unige.ch

Table of Contents

1.	Materials and Methods	S3
2.	Synthesis	S4
	2.1. Substrate Synthesis	S4
	2.2. Organocatalyst Synthesis	S5
3.	Electromicrofluidic Reactor Configuration	S9
	3.1. MWCNTs-Graphite Electrode	S10
4.	Product Analysis	S10
	4.1. Response Factors	S10
	4.2. Conversions and Yields	S12
5.	OEEF Catalyzed Cyclizations	S13
	5.1. Dependence on Current	S13
	5.2. Dependence on Flowrate	S17
6.	Organocatalyst Interfacing	S21
	6.1. Controls without Electromicrofluidics	S21
	6.2. Dependence on Current	S22
	6.3. Dependence on Flowrate	S27
	6.4. Dependence on Temperature	S28
	6.5. Dependence on Base	S30
7.	Hydroquinone Oxidation Control	S33
8.	GC Traces	S35
	8.1. Reference Compounds	S35
	8.2. Reaction Mixtures	S44
9.	Supporting References	S52
10.	NMR Spectra	S53

1. Materials and Methods

As in reference [S1], reagents for synthesis were purchased from Sigma-Aldrich, FluoroChem and Thermo-Fisher commercial suppliers. Anhydrous tetrahydrofuran, dimethylformamide, toluene, acetonitrile, methanol were purchased from Thermo Fischer Scientific and used as received. Anhydrous dichloromethane and triethylamine were distilled from calcium hydride. All moisture sensitive reactions were carried out under an argon atmosphere using oven-dried glassware. Analytical thin layer chromatography (TLC) was performed on silica gel 60 F254 (Merck, 0.2 mm). Column chromatography was carried out on silica gel 60 (SiliaFlash® P60, 230 – 400 mesh). All ^{1}H and ^{13}C NMR spectra were recorded (as indicated) on Bruker 400 MHz spectrometer at room temperature (25 °C) and are reported as chemical shifts (δ) in parts per million (ppm) relative to TMS ($\delta = 0$). Spin multiplicities were reported as a singlet (s), doublet (d), triplet (t) and quartet (q), with coupling constants (J) given in Hz, or multiplet (m). Broad signals are marked with br. ^{1}H and ^{13}C resonances were assigned with the aid of additional information from 1D and 2D NMR spectra (H,H-COSY, DEPT 135, HSQC, HMBC and ROESY). UV-vis absorption spectra were recorded using JASCO V-650 spectrometer. Gas chromatography (GC) was performed on Agilent 6850 Series gas chromatographs equipped with a split-mode capillary injection system and flame ionization detectors using HP-1 Methyl Siloxane column (30 m x 0.32 mm ID). Separation parameters: hold at 60 °C for 3 min, 10 °C·min^{-1} until 130 °C, then 20 °C·min^{-1} until 280 °C and hold at 280 °C for 15 min (Speed: 60 cm s^{-1} H_2, injector temperature: 300 °C). Flow electric-field experiments were performed similarly to those in ref. [S1] using a stand-alone Vapourtec Ion Electrochemical Reactor equipped with an Aim-TTi EX354RD Dual Power Supply from Thurlbym Thandar Instruments Ltd and Chemyx Fusion 100 Touch Syringe Pumps. Platinum (Pt) and graphite (Gr) electrodes were purchased from Goodfellow. MWCNT was from Sigma-Aldrich. The electrodes (5 x 5 cm^{2}) were separated by a 0.25 mm FEP spacer resulting in a reactor volume of 0.3 mL, with an exposed electrode surface area of

12 cm^2. Electric field catalysis experiments below room temperature were performed using EYELA Aluminum Block Cryostat PSL-2500B cooling system.

Abbreviations. DIPEA: *N,N*-Diisopropylethylamine; DMF: Dimethylformamide; DMSO: Dimethyl sulfoxide; EF: Electric field; EtOAc: Ethyl acetate; FEP: Fluorinated ethylene propylene; GC: Gas chromatography; Gr: Graphite electrode; HATU: Hexafluorophosphate azabenzotriazole tetramethyl uranium; MeOH: Methanol; MsCl: Methanesulfonyl chloride; MWCNTs: Multi-walled carbon nanotubes; PE: Petroleum ether (b.p. 40-65°); Py: Pyridine; Pt: Platinum electrode; TFA: Trifluoroacetic acid; THF: Tetrahydrofuran; TMSCl: Trimethylsilyl chloride; TosylCl: 4-Toluenesulfonyl chloride.

2. Synthesis

2.1. Substrate Synthesis

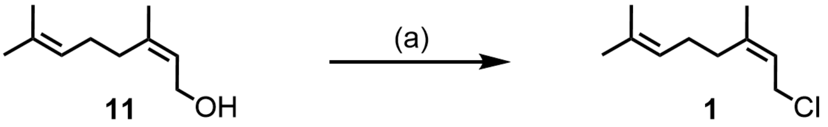

Scheme S1. Synthesis of neryl chloride **1** according to ref. [S2]. a) LiCl, MsCl, 2,4,6-collidine, DMF, 0 °C, 2 h, 59%.

Compound 1. Under inert atmosphere a mixture of **11** (2.3 mL, 13 mmol), LiCl (6.6 g, 16 mmol) and 2,4,6-collidine (7.0 mL, 52 mmol) in anhydrous DMF (70 mL) was cooled down to 0 °C. MsCl (2.0 mL, 26 mmol) was added dropwise into reaction mixture and then reaction was stirred for 2 h at 0 °C. Reaction mixture was poured over ice-water and the aqueous layer was extracted with pentane. The combined organic layer was washed with sat. copper sulfate solution to remove 2,4,6-collidine, and then brine. The organic layer was dried over Na_2SO_4, filtered and evaporated to dryness. Fractional distillation under vacuum (90 to 115 °C) of the residue afforded **1** (1.3 g, 59%) as a colorless oil. ^{1}H NMR (400 MHz, $CDCl_3$): 5.49 – 5.40 (m, 1H), 5.15 – 5.06 (m, 1H), 4.08 (dq, $^3J_{H-H}$ = 8.1 Hz, $^5J_{H-H}$ = 0.9 Hz, 2H), 2.18 – 2.04 (m, 4H), 1.77 (dt, $^4J_{H-H}$ = 1.7 Hz, $^4J_{H-H}$ = 0.9 Hz, 3H), 1.69

(s, 3H), 1.61 (s, 3H). Spectral data were in accordance with a literature.[S2] *Note:* Compound **1** used in this study contained a small (4-7%) impurity of α-terpineol depending on the batch.

2.2. Organocatalyst Synthesis

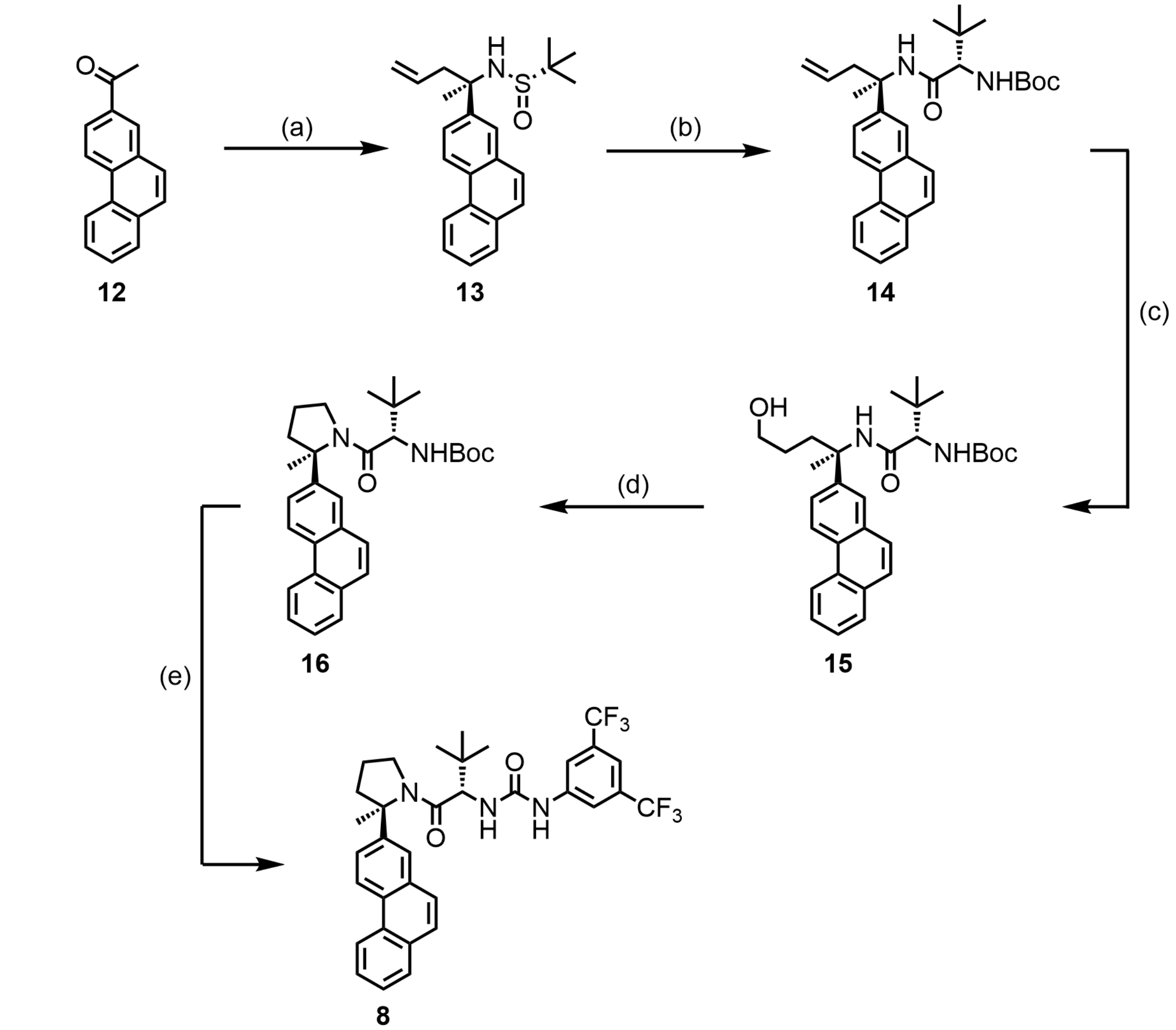

Scheme S2. Synthesis of catalyst **8** according to ref. [S2]. a) 1. $Ti(OEt)_4$, (S)-Ellman sulfinamide, THF, reflux, 12 h; 2. Zn, TMSCl, Allyl bromide, THF, 24 h, rt, 56%; b) 1. 4.0 M HCl in dioxane, MeOH, 0 °C, 1 h; 2. DIPEA, HATU, N-Boc-L-*tert*-leucine, CH_2Cl_2, rt, 24 h, 91%; c) 1. 9-BBN in hexane, THF, 0 °C, 8 h; 2. NaOH, H_2O_2, 24 h, rt, 71%; d) NaH, TosylCl, THF, rt, 24 h, 83%; e) 1. 4.0 M HCl in dioxane, 0 °C, 4 h; 2. Et_3N, 3,5-bis(trifluoromethyl)phenyl isocyanate, CH_2Cl_2, rt, 24 h, 90%.

Compound 13. Under inert atmosphere, to a solution of 2-acetylphenanthrene (5.0 g, 23 mmol) in anhydrous THF (50 mL) was added titanium ethoxide (11 mL, 50 mmol) and (S)-Ellman sulfinamide (3.0 g, 25 mmol). The reaction mixture was refluxed for 12 h. Then after cooling down to rt, the reaction mixture was passed through a short silica plug eluting with warm EtOAc (this was crucial to ensure that Ellman ketimine was soluble). The eluent was concentrated in vacuo and the residue was dissolved in anhydrous THF (130 mL). In a separate flask, to a suspension of zinc (1.6 g, 25 mmol) in anhydrous THF (50 mL) was added TMSCl (0.43 mL, 3.4 mmol) followed by allyl bromide (2.2 mL, 25 mmol). After stirring for 1 h at rt, the mixture was transferred into reaction mixture of ketimine. The reaction was stirred for 24 h at rt, then quenched with sat. aqueous NH_4Cl solution and extracted with EtOAc. The combined organic layer was washed with brine, dried over anhydrous Na_2SO_4, filtered and evaporated to dryness. The residue was purified by column chromatography on silica gel (PE/EA 1:1, R_f 0.26) to afford **13** (4.7 g, 56%) as a colorless solid. ^{1}H NMR (400 MHz, $CDCl_3$): 8.70 – 8.63 (m, 2H), 7.95 – 7.86 (m, 2H), 7.80 – 7.71 (m, 3H), 7.74 – 7.56 (m, 2H), 5.61 (ddt, $^3J_{H-H}$ = 17.5, 10.1, 7.3 Hz, 1H), 5.26 – 5.10 (m, 2H), 3.85 (s, 1H), 2.80 (d, $^3J_{H-H}$ = 7.3 Hz, 2H), 1.93 (s, 3H), 1.26 (s, 9H). Spectral data were in accordance with a literature.[S2]

Compound 14. Under inert atmosphere, to a solution of compound **13** (4.7 g, 13 mmol) in anhydrous MeOH (40 mL) was added 4.0 M HCl in dioxane (22 mL, 89 mmol) at 0 °C and was stirred for 1 h. The solvent was removed under reduced pressure and the residue was dissolved in anhydrous CH_2Cl_2 (60 mL). DIPEA (11 mL, 64 mmol) was added to a reaction mixture, followed by HATU (7.3 g, 19 mmol) and N-Boc-L-*tert*-leucine (3.6 g, 15 mmol) at 0 °C. The reaction mixture was allowed to reach rt while stirring for 24 h. Then the reaction was quenched with sat. aqueous NH_4Cl solution and extracted with EtOAc. The combined organic phase was washed with brine, dried over anhydrous Na_2SO_4 and evaporated to dryness. The residue was purified by column chromatography on silica gel (PE/Et_2O 2:1, R_f 0.27) to afford **14** (5.5 g, 91%) as a colorless solid. ^{1}H NMR (400 MHz, $CDCl_3$): 8.62 (t, $^3J_{H-H}$ = 8.6 Hz, 2H), 7.87 (dd, $^3J_{H-H}$ = 7.9 Hz, $^4J_{H-H}$ = 1.5 Hz, 1H), 7.80 (d, $^4J_{H-H}$ =

2.2 Hz, 1H), 7.75 – 7.66 (m, 2H), 7.66 – 7.60 (m, 2H), 7.61 – 7.53 (m, 1H), 6.14 (s, 1H), 5.64 (ddt, $^{3}J_{H\text{-}H}$ = 17.3, 10.1, 7.3 Hz, 1H), 5.26 – 5.14 (m, 3H), 3.85 (d, $^{3}J_{H\text{-}H}$ = 9.5 Hz, 1H), 2.83 (dd, $^{2}J_{H\text{-}H}$ = 13.7 Hz, $^{3}J_{H\text{-}H}$ = 7.3 Hz, 1H), 2.63 (dd, $^{2}J_{H\text{-}H}$ = 13.7 Hz, $^{3}J_{H\text{-}H}$ = 7.3 Hz, 1H), 1.92 (s, 3H), 1.49 (s, 9H), 1.03 (s, 9H). Spectral data were in accordance with a literature.[S2]

Compound 15. Under inert atmosphere to a mixture of **14** (5.5 g, 12 mmol) in anhydrous THF (45 mL) was added a solution of 9-BBN in hexane solution (0.40 M, 38 mL, 15 mmol) at 0 °C and the mixture was stirred for 8 h. Then the aqueous 2.0 M NaOH solution (58 mL, 120 mmol) was added, followed by dropwise addition of H_2O_2 (50 wt.%, 18 mL). The reaction was allowed to reach rt while stirring for 24 h, then was diluted with brine and extracted with Et_2O. Combined organic phase was washed with brine, dried over anhydrous Na_2SO_4 and evaporated to dryness. The residue was purified by column chromatography on silica gel (Et_2O, R_f 0.30) to afford **15** (4.1 g, 71%) as a yellow oil. ^{1}H NMR (400 MHz, $CDCl_3$): 8.65 – 8.54 (m, 2H), 7.86 (dd, $^{3}J_{H\text{-}H}$ = 7.8 Hz, $^{4}J_{H\text{-}H}$ = 1.8 Hz, 1H), 7.78 (d, $^{4}J_{H\text{-}H}$ = 1.8 Hz, 1H), 7.73 – 7.65 (m, 2H), 7.65 – 7.52 (m, 3H), 7.02 – 6.97 (m, 1H), 5.23 (d, $^{3}J_{H\text{-}H}$ = 9.8 Hz, 1H), 3.83 (d, $^{3}J_{H\text{-}H}$ = 9.8 Hz, 1H), 3.61 – 3.53 (m, 2H), 2.68 (s, 1H), 2.22 – 2.06 (m, 1H), 2.05 – 1.93 (m, 1H), 1.90 (s, 3H), 1.48 (s, 9H), 1.45 – 1.36 (m, 2H), 1.04 (s, 9H). Spectral data were in accordance with a literature.[S2]

Compound 16. Under inert atmosphere, a solution of compound **15** (5.1 g, 10 mmol) in anhydrous THF (15 mL) was added dropwise into suspension of NaH 60% (1.7 g, 42 mmol) in anhydrous THF (50 mL) at 0 °C. After stirring for 30 min, the solution of TosylCl (2.4 g, 13 mmol) in anhydrous THF (15 mL) was added dropwise into reaction mixture. The reaction was stirred for 24 h allowing to reach rt. Then the reaction mixture was diluted with brine and extracted with Et_2O. The combined organic phase was washed with brine, dried over anhydrous Na_2SO_4 and evaporated to dryness. The residue was purified by column chromatography on silica gel (PE/Et_2O 2:1 R_f 0.40) to afford **16** (4.1 g, 83%) as an amorphous solid. ^{1}H NMR (400 MHz, $CDCl_3$): 8.63 – 8.51 (m, 2H), 7.85 (dd, $^{3}J_{H\text{-}H}$ = 7.8 Hz, $^{4}J_{H\text{-}H}$ = 1.5 Hz, 1H), 7.73 – 7.64 (m, 3H), 7.64 – 7.59 (m, 1H), 7.58 – 7.52

(m, 1H), 7.47 (dd, $^{3}J_{H\text{-}H}$ = 8.7, $^{4}J_{H\text{-}H}$ = 2.1 Hz, 1H), 5.17 (d, $^{3}J_{H\text{-}H}$ = 9.9 Hz, 1H), 4.41 (d, $^{3}J_{H\text{-}H}$ = 9.9 Hz, 1H), 4.31 (dt, $^{2}J_{H\text{-}H}$ = 9.8 Hz, $^{3}J_{H\text{-}H}$ = 6.2 Hz, 1H), 3.89 (dt, $^{2}J_{H\text{-}H}$ = 9.8 Hz, $^{3}J_{H\text{-}H}$ = 7.4 Hz, 1H), 2.19 – 2.07 (m, 2H), 2.04 (s, 3H), 2.01 – 1.90 (m, 2H), 1.54 (s, 9H), 1.05 (s, 9H). Spectral data were in accordance with a literature.[S2]

Compound 8. Under inert atmosphere to a solution of compound **16** (4.1 g, 8.6 mmol) in anhydrous CH_2Cl_2 (60 mL) was added 4.0 M HCl in dioxane (86 mL, 350 mmol) dropwise over 5 min at 0 °C. After stirring for 4 h, the reaction mixture was carefully quenched with sat. $NaHCO_3$ aqueous solution and extracted with Et_2O. The combined organic phase was dried over anhydrous Na_2SO_4, filtered and evaporated to dryness. The resulting residue was dissolved in anhydrous CH_2Cl_2 (60 mL), followed by addition of anhydrous Et_3N (3.6 mL, 26 mmol) and 3,5-bis(trifluoromethyl)phenyl isocyanate (1.9 mL, 11 mmol). The reaction mixture was stirred 24 h at rt. Then the reaction was diluted with brine and extracted with CH_2Cl_2. The combined organic phase was washed with brine, dried over anhydrous Na_2SO_4, filtered and evaporated to dryness. The residue was purified by column chromatography on silica gel ($CHCl_3$/EA 20:1, R_f 0.24) to afford **8** (4.9 g, 90%) as an amorphous solid. ^{1}H NMR (400 MHz, $CDCl_3$): 8.43 – 8.24 (m, 2H), 7.93 (br.s, 1H), 7.82 – 7.72 (m, 1H), 7.64 – 7.50 (m, 4H), 7.49 – 7.42 (m, 2H), 7.40 – 7.32 (m, 3H), 6.06 (br.s, 1H), 4.76 (d, $^{3}J_{H\text{-}H}$ = 9.6 Hz, 1H), 4.32 (dt, $^{2}J_{H\text{-}H}$ = 10.1 Hz, $^{3}J_{H\text{-}H}$ = 6.8 Hz, 1H), 3.95 (dt, $^{2}J_{H\text{-}H}$ = 10.1 Hz, $^{3}J_{H\text{-}H}$ = 6.8 Hz, 1H), 2.15 (t, $^{3}J_{H\text{-}H}$ = 6.8 Hz, 2H), 2.05 (s, 3H), 2.02 – 1.90 (m, 2H), 1.09 (s, 9H). Spectral data were in accordance with a literature.[S2]

3. Electromicrofluidic Reactor Configuration

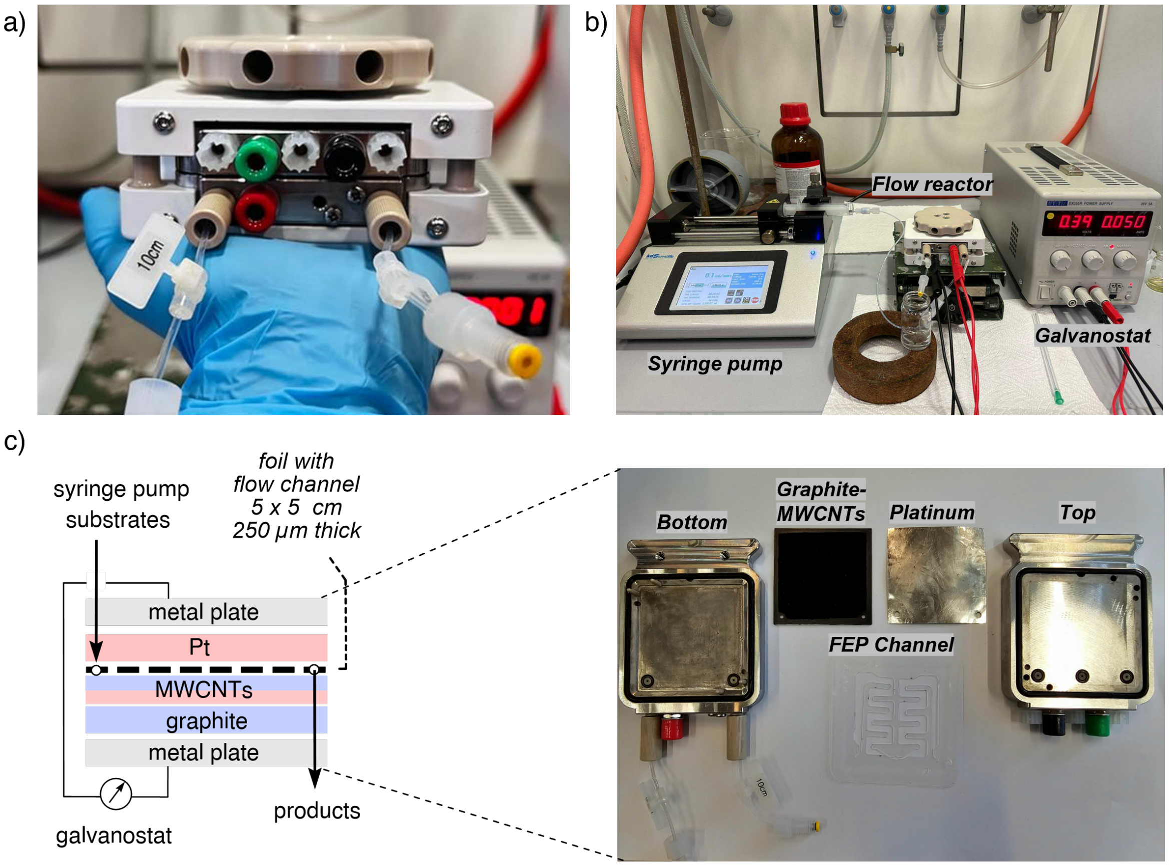

Figure S1. a) An assembled Vapourtec Ion electrochemical flow reactor; b) set up of the system; c) schematic representation of the flow system (left) and its components (right).

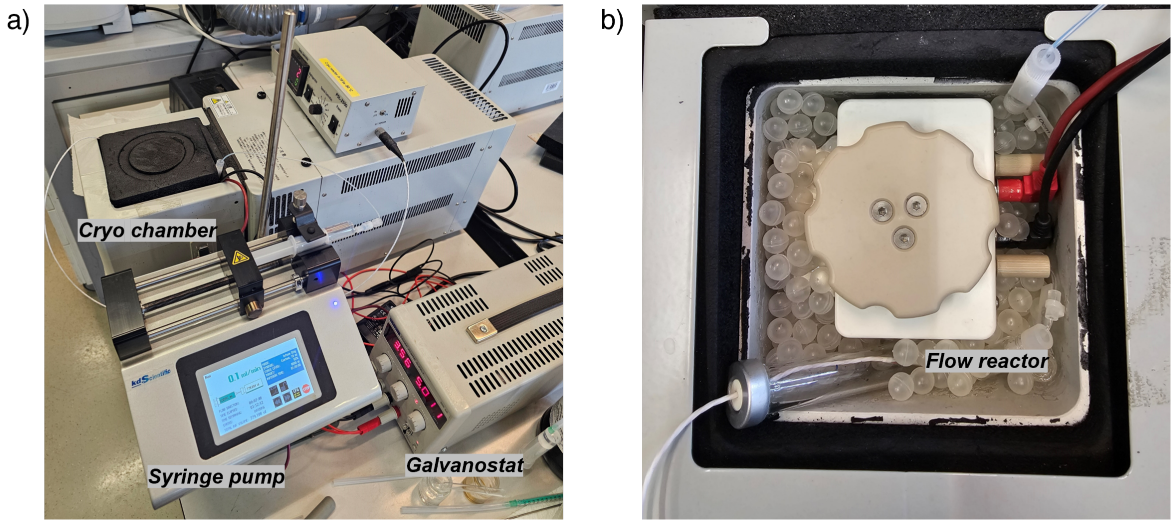

Figure S2. a) Cryo chamber used for the control of reactor's temperature; b) flow reactor placed into the cryo chamber.

3.1. MWCNTs-Graphite Electrode

Coated electrode was prepared as described in the reference [S1] and [S3]. MWCNTs (23.0 mg) were suspended in DMF (11.5 mL) and the mixture was sonicated for 30 minutes to obtain a 2.0 mg mL^{-1} black suspension. The electrode was coated by drop casting MWCNTs suspension on the freshly polished graphite electrode and the solvent was allowed to dry in air for 15 h. Then the surface was rinsed with distilled water and dried using the pre-heated oven at 130 °C.

4. Product Analysis

4.1. Response Factors

Conversion and yields of monoterpenes were calculated based on previously reported method by Tiefenbacher group.[S4] Products were identified by comparing retention times in GC with commercially available samples of known monoterpenes (Figure S12-27). GC-response factors to *n*-decane as internal standard (IS) were calculated for all identified monoterpenes to estimate conversion and yields of products. Cyclic monoterpene products in the prepared samples were within the same range as the observed concentrations in cyclization reactions, whereas *n*-decane, used as internal standard, had the exact same concentration. The response factors were calculated according to the following Equation [S1], where RF – response factor, A_x – area of analyte, A_{IS} – area of internal standard, C_x – concentration of analyte, C_{IS} – concentration of internal standard, and are listed in Table S1.

$$RF = (A_x \times C_{IS})/(A_{IS} \times C_x), \qquad [S1]$$

To prepare internal standard and monoterpene analytes samples for GC analysis, 20 µL of *n*-decane stock solution (0.25 M) in Et_2O, and 5.0 µL, 10 µL or 20 µL of stock solution of monoterpenes (0.50 M) in CH_3CN were diluted to a total volume of 0.10 mL in CH_3CN to obtain solutions of monoterpenes with concentrations 0.025 M, 0.05 M and 0.10 M, respectively. Then 50 µL of each newly obtained solution was diluted in Et_2O (1.0 mL), filtered through layer of neutral Al_2O_3 and subjected to GC for analysis.

Table S1. Determined response factors for substrates and cyclic monoterpene products.

Analyte	C_x/C_{IS}[a]	A_x/A_{IS}[b]	RF[c]	Average
	0.5	0.454	0.91	
Neryl chloride	1.0	0.878	0.88	0.89
	2.0	1.770	0.89	
	0.5	0.377	0.75	
α-Terpinene	1.0	0.753	0.75	0.75
	2.0	1.467	0.73	
	0.5	0.436	0.87	
Limonene	1.0	0.874	0.87	0.88
	2.0	1.762	0.88	
	0.5	0.425	0.85	
Terpinolene	1.0	0.850	0.85	0.85
	2.0	1.723	0.86	
	0.5	0.481	0.96	
γ-Terpinene	1.0	0.973	0.97	0.96
	2.0	1.893	0.95	
	0.5	0.593	1.19	
α-Terpineol	1.0	1.224	1.22	1.19
	2.0	2.349	1.17	
	0.5	0.396	0.79	
α-Terpinyl chloride	1.0	0.798	0.80	0.79
	2.0	1.574	0.79	
p-Cymene	0.5	0.481	0.96	0.95

1.0	0.970	0.97
2.0	1.837	0.92

[a] Ratio of the concentration of the analyte (C_x) to the concentration of the internal standard (C_{IS}) in an analyzed sample. [b] Ratio of the peak area of the analyte (A_x) to the peak area of internal standard (A_{IS}) in an analyzed sample. [c] Response factor.

4.2. Conversions and Yields

Conversions and yields were calculated by utilizing the following Equations [S2]-[S6], where $n(sm)_0$ – initial amount (relative) of starting material in reaction mixture, $n(sm)_n$ – amount of starting material in collected sample after the reaction, $n(p)_n$ - amount of cyclic product in collected sample after the reaction, $(A_{sm})_n$ – area of starting material in the n-th collected sample, $(A_{IS})_n$ – area of internal standard in the n-th collected sample, $(A_p)_n$ – area of product in the n-th collected sample, RF_{sm} – response factor of starting material, RF_p – response factor of product. The calculated yields and conversions were plotted against current or flowrate (Figure S3-S10).

At $t = 0$:

$$n(sm)_0 = \frac{(A_{sm})_0/(A_{IS})_0}{RF_{sm}} \qquad [S2]$$

for all the of rest measurements:

$$n(sm)_n = \frac{(A_{sm})_n/(A_{IS})_n}{RF_{sm}} \qquad [S3]$$

$$conversion(sm) = (n(sm)_0 - n(sm)_n)/n(sm)_0 \qquad [S4]$$

$$n(p)_n = \frac{(A_p)_n/(A_{IS})_n}{RF_p} \qquad [S5]$$

$$yield = n(p)_n/\, n(sm)_0 \qquad [S6]$$

5. OEEF Catalyzed Cyclizations

5.1. Dependence on Current

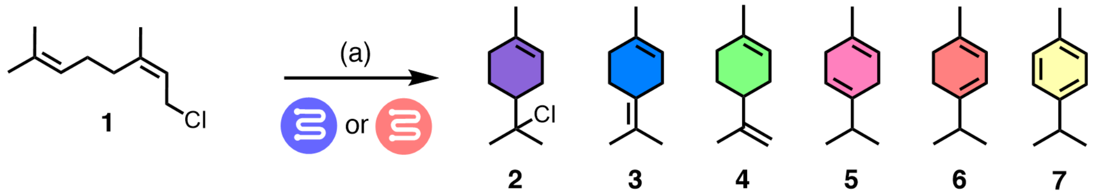

Scheme S3. OEEF catalyzed neryl chloride **1** cyclization in flow. a) EF, 100 mM, toluene, 15 μL·min^{-1}.

Electric-field catalysis in flow was performed in an undivided cell using a Vapourtec Ion Electrochemical Reactor (FEP spacer = 0.25 mm, reactor volume = 0.30 mL) in galvanostatic mode, employing a platinum electrode and a graphite electrode coated with MWCNTs (active surface area = 12 cm^2 for each electrode). A stock solution of neryl chloride **1** (100 mM, 1.0 equiv) and *n*-decane (0.5 equiv) in toluene (6 mL) was pumped with a constant flowrate of 15 μL·min^{-1} into the electrochemical reactor to screen different current conditions at ambient temperature. The first one and a half reactor volumes (0.45 mL) were disposed to ensure a steady state of the system had been reached. After collection for a defined period (13 min), 50 μL of the reaction mixture was diluted in 1.0 mL Et_2O, filtered through layer of Al_2O_3 and subjected to GC analysis. Conversions and yields were calculated from obtained GC traces by utilizing Equations [S2]-[S6].

For positive electric field catalyzed monoterpene cyclization, current was applied by employing a platinum electrode as the cathode and a graphite electrode coated with MWCNTs as the anode. For negative electric field catalyzed monoterpene cyclization, current was applied in opposite direction by employing a platinum electrode as the anode and graphite electrode coated with MWCNTs as the cathode.

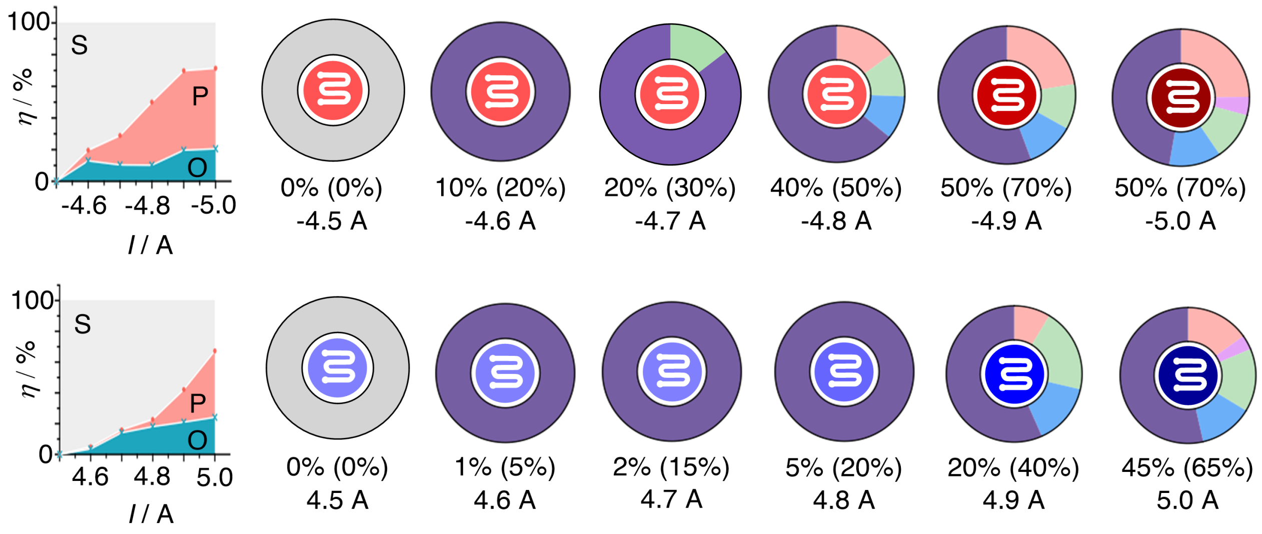

Figure S3. Cyclization of neryl chloride **1** in toluene catalyzed by negative (top) and positive (bottom) electric fields at constant flow (v = 15 μL·min^{-1}) on multiwalled carbon nanotubes in electromicrofluidic reactor. Results are reported in Tables S2, S3 and GC traces in Figures S29, S30. Substrate (grey), product (red) and unidentified products (teal) plotted as a function of the applied current and doughnut charts for product distribution with total yield (conversion) below each chart.

Table S2. Monoterpene cyclizations catalyzed by negative electric fields on multiwalled carbon nanotubes in electromicrofluidic reactors.[a]

Entry	I [A][b]	V [V][c]	η [%][d]	P [%][e]					O [%][f]	S [%][g]	P+O [%][h]
				**2**	**3**	**4**	**5**	**6**			
1	-4.5	-3.4	0	0	0	0	0	0	0	100	0
2	-4.6	-3.4	7	7	0	0	0	0	13	80	20
3	-4.7	-3.6	19	16	0	3	0	0	10	71	29
4	-4.8	-4.1	40	26	4	4	0	6	10	50	50
5	-4.9	-3.9	51	28	6	6	0	11	19	30	70
6	-5.0	-3.9	51	24	6	6	2	13	20	29	71

[a] Conditions: Gr-MWCNTs cathode, Pt anode, 15 $\mu L \cdot min^{-1}$ flowrate, toluene. [b] Current applied, in ampere. [c] Measured voltage, in volt, fluctuated within ± 100 mV range. [d] Total yield of main cyclic monoterpenes. [e] Yields of assigned cyclic products after one passage through the electromicrofluidic reactor. [f] Yields of other unidentified products, including loss of volatile materials and decomposition. [g] Substrate left after one passage through the electromicrofluidic reactor. [h] Total conversion. Conversions and yields were calculated from obtained GC traces (Figure S29) by utilizing Equations [S2]-[S6]. Summary of corresponding data presented in Figure S3.

Table S3. Monoterpene cyclizations catalyzed by positive electric fields on multiwalled carbon nanotubes in electromicrofluidic reactors.[a]

Entry	I [A][b]	V [V][c]	η [%][d]	P [%][e]					O [%][f]	S [%][g]	P+O [%][h]
				**2**	**3**	**4**	**5**	**6**			
1	4.5	3.6	0	0	0	0	0	0	0	100	0
2	4.6	3.6	1	1	0	0	0	0	4	95	5
3	4.7	3.8	2	2	0	0	0	0	14	84	16
4	4.8	4.2	5	5	0	0	0	0	18	77	23
5	4.9	4.4	21	12	3	4	0	2	21	58	42
6	5.0	4.5	43	23	5	7	1	7	24	33	67

[a] Conditions: Pt cathode, Gr-MWCNTs anode, 15 $\mu L \cdot min^{-1}$ flowrate, toluene. [b] Current applied, in ampere. [c] Measured voltage, in volt, fluctuated within ± 100 mV range. [d] Total yield of main cyclic monoterpenes. [e] Yields of assigned cyclic products after one passage through the electromicrofluidic reactor. [f] Yields of other unidentified products, including loss of volatile materials and decomposition. [g] Substrate left after one passage through the electromicrofluidic reactor. [h] Total conversion. Conversions and yields were calculated from obtained GC traces (Figure S30) by utilizing the Equations [S2]-[S6]. Summary of corresponding data presented in Figure S3.

5.2. Dependence on Flowrate

Electric-field catalysis in flow was performed in an undivided cell using a Vapourtec Ion Electrochemical Reactor (FEP spacer = 0.25 mm, reactor volume = 0.30 mL) in galvanostatic mode, employing a platinum electrode and a graphite electrode coated with MWCNTs (active surface area = 12 cm^2 for each electrode). The flow reactor was placed in a cryo chamber to ensure 18 ^{o}C temperature. A stock solution of neryl chloride **1** (100 mM, 1.0 equiv) and *n*-decane (0.5 equiv) in anhydrous toluene (8.0 mL) was pumped at constant current I (5.0 A) into an electrochemical reactor to screen different flowrate conditions at 18 ^{o}C. The first one and a half reactor volumes (0.45 mL) were disposed to ensure a steady state of the system had been reached. After collection for a defined period, 50 μL of the reaction mixture was diluted in 1.0 mL Et_2O, filtered through layer of Al_2O_3 and subjected to GC analysis.

For positive electric field catalyzed monoterpene cyclization, current was applied by employing a platinum electrode as the cathode and a graphite electrode coated with MWCNTs as the anode. For negative electric field catalyzed monoterpene cyclization, current was applied in opposite direction by employing a platinum electrode as the anode and graphite electrode coated with MWCNTs as the cathode.

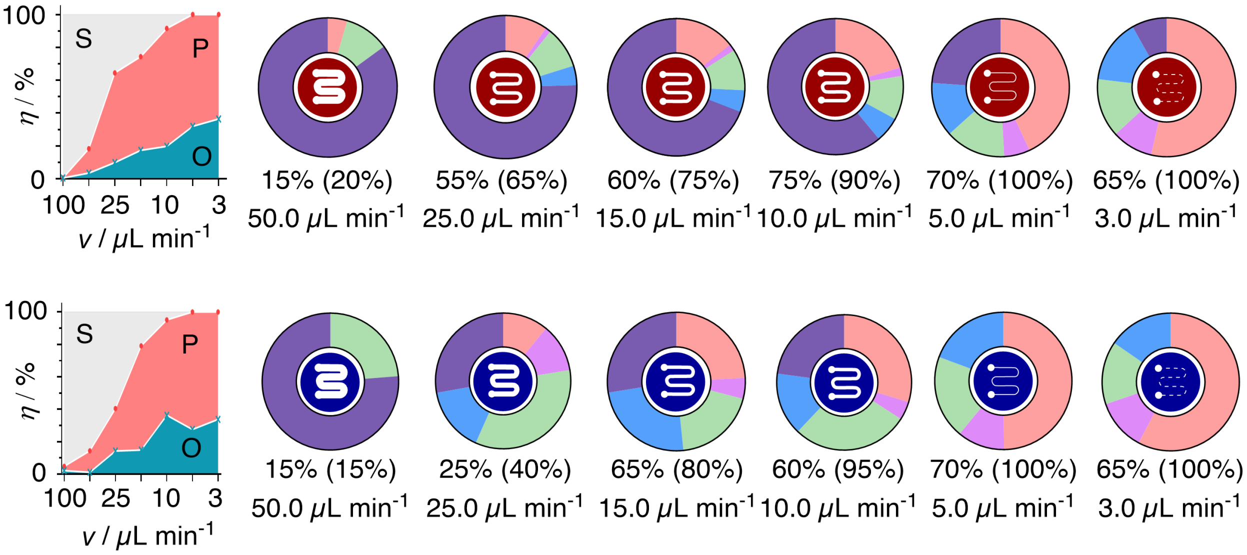

Figure S4. Cyclization of neryl chloride **1** in toluene catalyzed by negative (top) and positive (bottom) electric fields at constant current (I = 5.0 A) on multiwalled carbon nanotubes in electromicrofluidic reactor. Results are reported in Tables S4 and S5 and GC traces in Figures S31, S32. Substrate (grey), product (red) and unidentified products (teal) plotted as a function of flowrate and doughnut charts for product distribution with total yield (conversion) below each chart. In doughnut charts p-cymene **7** yield reported together with α-terpinene yield.

Table S4. Optimization of flowrate for monoterpene cyclizations catalyzed by negative electric fields on multiwalled carbon nanotubes in electromicrofluidic reactors.[a]

Entry	v [μL·min^{-1}][b]	V [V][c]	η [%][d]	P [%][e]						O [%][f]	S [%][g]	P+O [%][h]
				**2**	**3**	**4**	**5**	**6**	**7**			
1	100	-3.8	0	0	0	0	0	0	0	1	99	1
2	50	-4.4	17	14	0	2	0	1	0	1	82	18
3	25	-4.1	55	41	3	5	1	5	0	10	35	65
4	15	-3.7	61	42	3	6	1	8	1	14	25	75
5	10	-4.1	73	45	4	8	1	14	1	19	8	92
6	5	-4.6	70	17	9	10	4	28	2	30	0	100
7	3	-4.7	66	6	10	9	6	33	2	34	0	100

[a] Conditions: Gr-MWCNTs cathode, Pt anode, I = -5.0 A, toluene. [b] Flowrate applied, in *micro*liters per minute. [c] Measured voltage, in volt, fluctuated within ± 100 mV range. [d] Total yield of main cyclic monoterpenes. [e] Yields of assigned cyclic products after one passage through the electromicrofluidic reactor. [f] Yields of other unidentified products, including loss of volatile materials and decomposition. [g] Substrate left after one passage through the electromicrofluidic reactor. [h] Total conversion. Conversions and yields were calculated from obtained GC traces (Figure S31) by utilizing Equations [S2]-[S6]. Summary of corresponding data presented in Figure S4.

Table S5. Optimization of flowrate for monoterpene cyclizations catalyzed by positive electric fields on multiwalled carbon nanotubes in electromicrofluidic reactors.[a]

Entry	v [μL·min^{-1}][b]	V [V][c]	η [%][d]	P [%][e]						O [%][f]	S [%][g]	P+O [%][h]
				**2**	**3**	**4**	**5**	**6**	**7**			
1	100	3.7	3	3	0	0	0	0	0	2	95	5
2	50	3.9	13	10	0	3	0	0	0	1	86	14
3	25	4.0	26	7	4	9	3	3	0	14	60	40
4	15	4.1	64	18	15	12	3	15	1	15	21	79
5	10	4.6	59	13	9	16	3	16	2	36	5	95
6	5	5.1	73	0	14	14	8	29	8	27	0	100
7	3	5.1	66	0	10	10	8	31	7	34	0	100

[a] Conditions: Pt cathode, Gr-MWCNTs anode, I = 5.0 A, toluene. [b] Flowrate applied, in *micro*liters per minute. [c] Measured voltage, in volt, fluctuated within ± 100 mV range. [d] Total yield of main cyclic monoterpenes. [e] Yields of assigned cyclic products after one passage through the electromicrofluidic reactor. [f] Yields of other unidentified products, including loss of volatile materials and decomposition. [g] Substrate left after one passage through the electromicrofluidic reactor. [h] Total conversion. Conversions and yields were calculated from obtained GC traces (Figure S32) by utilizing Equations [S2]-[S6]. Summary of corresponding data presented in Figure S4.

6. Organocatalyst Interfacing

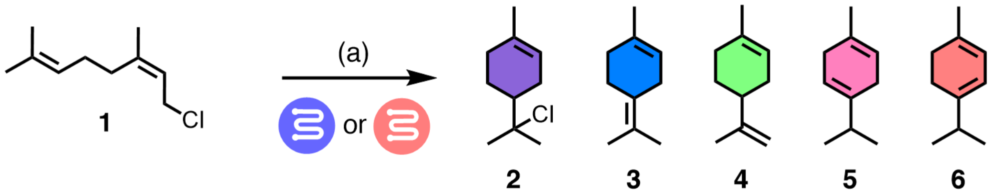

Scheme S4. OEEF catalyzed monoterpene cyclization in flow. a) Organocatalyst **8**, EF, 100 mM, toluene, 10 μL·min^{-1}.

6.1. Controls without Electromicrofluidics

A mixture of neryl chloride **1** (17 mg, 0.10 mmol), urea catalyst **8** (63 mg, 0.10 mmol) and n-decane (9.7 μL, 0.050 mmol) in anhydrous toluene (1.0 mL) were stirred for 24 h in the dark. After 5 min, 30 min, 1 h, 2 h, 4 h, 6 h, 9 h, 22 h, and 24 h, 50 μL of reaction mixture was taken and diluted in 1.0 mL Et_2O in separate vials, filtered through layer of Al_2O_3 and subjected to GC analysis. Conversions and yields were calculated from obtained GC traces by utilizing the Equations [S2]-[S6]. Summary of corresponding data presented in Figure S5 and Table S6.

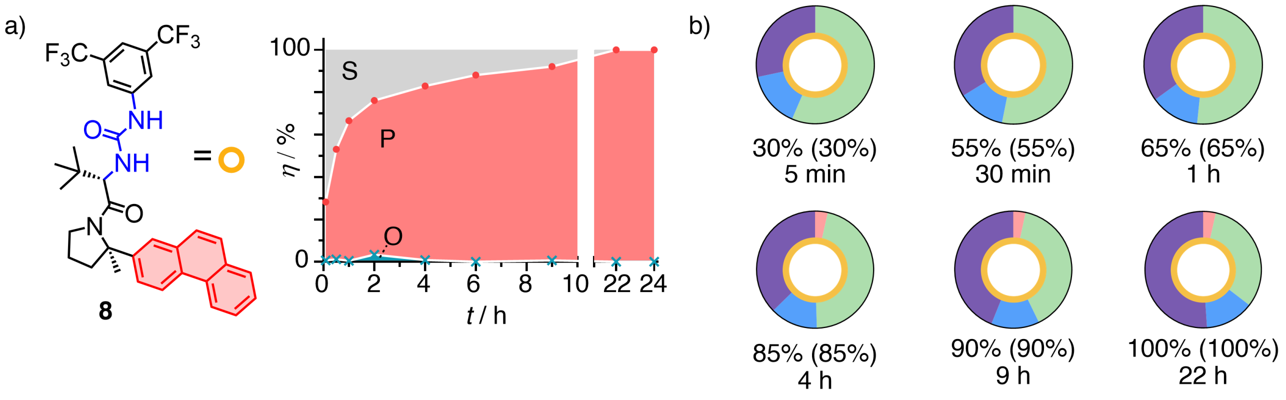

Figure S5. Control experiment of neryl chloride **1** (100 mM) mixed with organocatalyst **8** (1.0 equiv) in bulk solution without EF (a) and product distribution over time with total yield (conversion) below each chart (b). Results are reported in Tables S6 and GC traces in Figure S33. Substrate (grey), product (red) and unidentified products (teal) plotted as a function of time and doughnut charts for product distribution with total yield (conversion) below each chart.

Table S6. Control experiment results of neryl chloride **1** mixed with organocatalyst **8** without electromicrofluidics.[a]

Entry	t [h][a]	η [%][c]	P [%][d]					O [%][e]	S [%][f]	P+O [%][g]
			**2**	**3**	**4**	**5**	**6**			
1	0.08	28	8	4	16	0	0	0	72	28
2	0.5	53	18	7	28	0	0	0	47	53
3	1	67	23	9	35	0	0	0	33	67
4	2	75	27	10	38	0	0	1	24	76
5	4	84	31	11	39	0	3	0	16	84
6	6	88	36	12	37	0	3	0	12	88
7	9	93	41	12	37	0	3	0	7	93
8	22	100	53	13	31	0	3	0	0	100
9	24	100	58	13	26	0	3	0	0	100

[a] Conditions: 100 mM, organocatalyst **8** (1.0 equiv), rt, toluene. [b] Reaction time. [c] Total yield of main cyclic monoterpenes. [d] Yields of cyclic products. [e] Other unidentified products, including loss of volatile materials and decomposition. [f] Substrate. [g] Total conversion. Conversions and yields were calculated from obtained GC traces (Figure S33) by utilizing Equations [S2]-[S6]. Summary of corresponding data presented in Figure S5.

6.2. Dependence on Current

Electric-field assisted catalysis in flow was performed in an undivided cell using a Vapourtec Ion Electrochemical Reactor (FEP spacer = 0.25 mm, reactor volume = 0.30 mL) in galvanostatic mode, employing a platinum electrode and a graphite electrode coated with MWCNTs (active surface

area = 12 cm^2 for each electrode). Stock solutions of neryl chloride **1** (100 mM, 1.0 equiv) and *n*-decane (0.5 equiv) in anhydrous toluene (4.0 mL) and organocatalyst **8** (0.1 – 1.0 equiv) in anhydrous toluene (4.0 mL) were placed in separate syringes and pumped through T-mixer into the electrochemical reactor with a constant flowrate of 10 $\mu L \cdot min^{-1}$ to screen different current conditions at ambient temperature. The first one and a half reactor volumes (0.45 mL) were disposed to ensure a steady state of the system had been reached. After collection for a defined period (5 min) into a vial with 1.0 mL Et_2O, the reaction mixture was filtered through layer of Al_2O_3 and subjected to GC analysis. Conversions and yields were calculated from obtained GC traces by utilizing Equations [S2]-[S6].

For positive electric field catalyzed monoterpene cyclization, current was applied by employing a platinum electrode as the cathode and a graphite electrode coated with MWCNTs as the anode. For negative electric field catalyzed monoterpene cyclization, current was applied in opposite direction by employing a platinum electrode as the anode and graphite electrode coated with MWCNTs as the cathode.

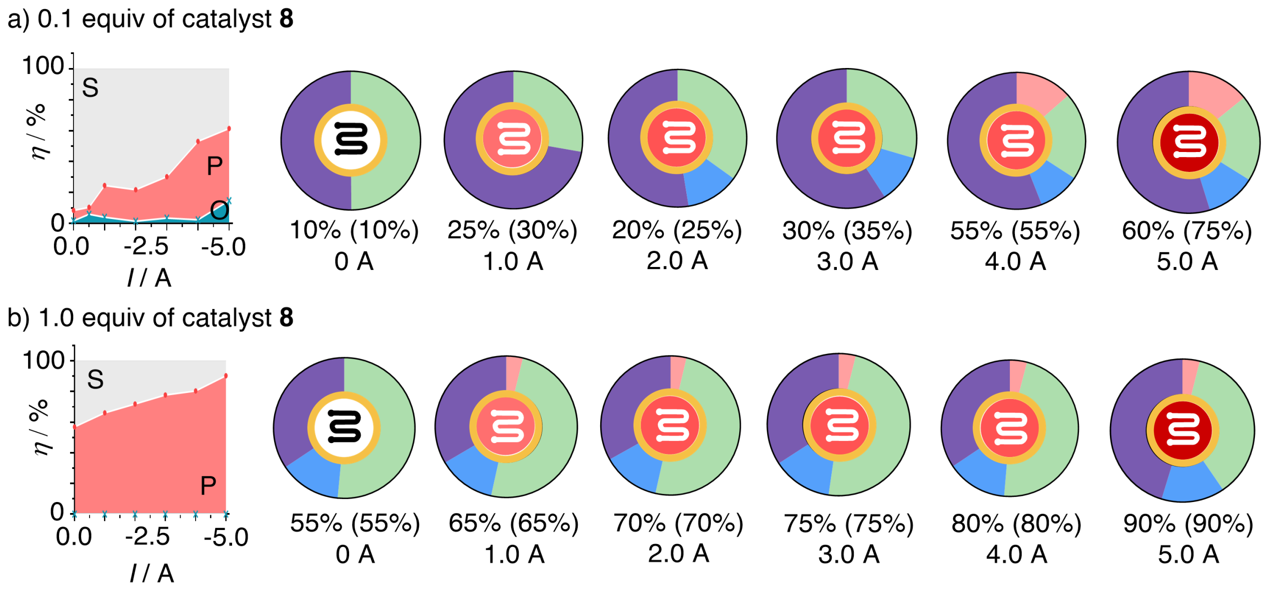

Figure S6. Interfacing organocatalyst **8** (0.1 equiv, a; and 1.0 equiv, b) with negative electric fields at constant flow (v = 10 μL·min^{-1}) to facilitate monoterpene cyclization on multiwalled carbon nanotubes in electromicrofluidic reactor. Results are reported in Table S7 entries 6 and 7 and GC traces in Figure S34. Substrate (grey), product (red) and unidentified products (teal) plotted as a function of the applied current and doughnut charts for product distribution with total yield (conversion) below each chart.

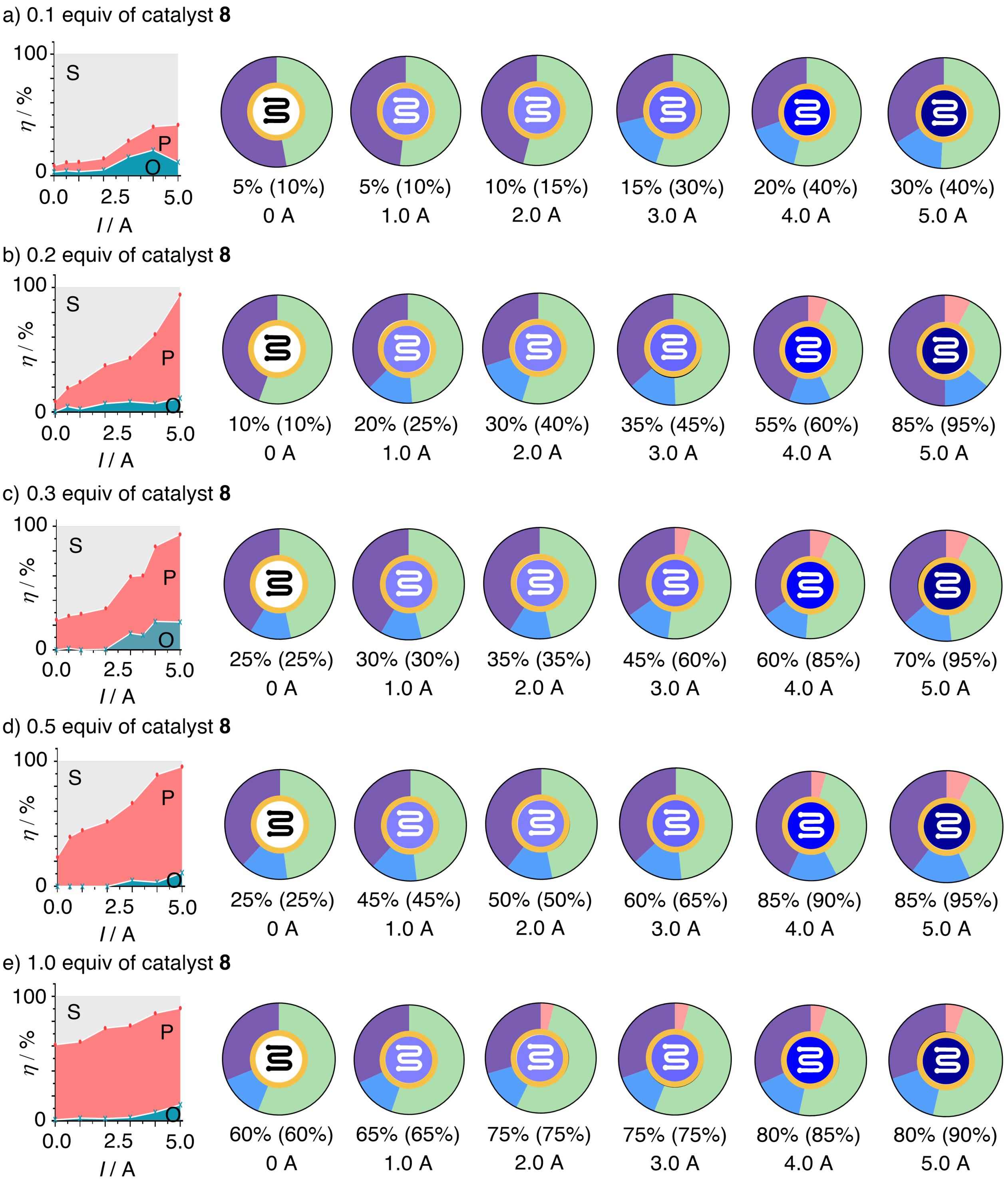

Figure S7. Interfacing organocatalyst **8** (0.1 – 1.0 equiv; (a-e)) with positive electric fields at constant flow (v = 10 µL·min^{-1}) to facilitate monoterpene cyclization on multiwalled carbon nanotubes in electromicrofluidic reactor. Results are reported in Table S7 entries 1-5 and GC traces in Figure S35.

Substrate (grey), product (red) and unidentified products (teal) plotted as a function of the applied current and doughnut charts for product distribution with total yield (conversion) below each chart.

Table S7. Monoterpene cyclizations catalyzed by positive or negative electric fields in presence of catalyst **8** on multiwalled carbon nanotubes in electromicrofluidic reactors.[a]

Entry	**8** [mol%]	I [A][b]	V [V][c]	η [%][d]	P [%][e]					O [%][f]	S [%][g]	P+O [%][h]
					**2**	**3**	**4**	**5**	**6**			
1	10	5.0	4.4	32	11	5	16	0	0	10	58	42
2	20	5.0	4.5	84	42	12	24	0	6	10	6	94
3	30	5.0	4.7	71	26	11	29	0	5	23	6	94
4	50	5.0	4.7[i]	85	34	15	30	0	6	11	4	96
5	100	5.0	4.2	80	24	13	39	0	4	11	9	91
6	10	-5.0	-4.0[i]	62	34	7	12	0	9	14	24	76
7	100	-5.0	-3.9	90	41	12	33	0	4	0	10	90

[a] Conditions: 10 µL·min^{-1} flowrate, toluene. [b] Current applied, in ampere, using Pt cathode, Gr-MWCNTs anode (for 5.0 A), or Pt cathode, Gr-MWCNTs anode (for -5.0 A). [c] Measured voltage, in volt. [d] Total yield of main cyclic monoterpenes. [e] Yields of assigned cyclic products after one passage through the electromicrofluidic reactor. [f] Yields of other unidentified products, including loss of volatile materials and decomposition. [g] Substrate left after one passage through the electromicrofluidic reactor. [h] Total conversion. [i] Voltage fluctuated within ± 300 mV range. Conversions and yields were calculated from obtained GC traces (Figures S34-S36). Summary of corresponding data presented in Figures S6 and S7.

6.3. Dependence on Flowrate

Electric-field assisted catalysis in flow was performed in an undivided cell using a Vapourtec Ion Electrochemical Reactor (FEP spacer = 0.25 mm, reactor volume = 0.30 mL) in galvanostatic mode, employing a platinum electrode and a graphite electrode coated with MWCNTs (active surface area = 12 cm^2 for each electrode). Stock solutions of neryl chloride **1** (100 mM, 1.0 equiv) and *n*-decane (0.5 equiv) in anhydrous toluene (4.0 mL), and organocatalyst **8** (0.2 equiv) in anhydrous toluene (4.0 mL) were placed in separate syringes and pumped through T-mixer into the electrochemical reactor at constant current *I* (2.0 A) to screen different flowrate conditions at ambient temperature. The first one and a half reactor volumes (0.45 mL) were disposed to ensure a steady state of the system had been reached. After collection for a defined period (5 min) into a vial with 1.0 mL Et_2O, the reaction mixture was filtered through layer of Al_2O_3 and subjected to GC analysis. Conversions and yields were calculated from obtained GC traces by utilizing the Equations [S2]-[S6].

For positive electric field catalyzed monoterpene cyclization, current was applied by employing a platinum electrode as the cathode and a graphite electrode coated with MWCNTs as the anode. For negative electric field catalyzed monoterpene cyclization, current was applied in opposite direction by employing a platinum electrode as the anode and graphite electrode coated with MWCNTs as the cathode.

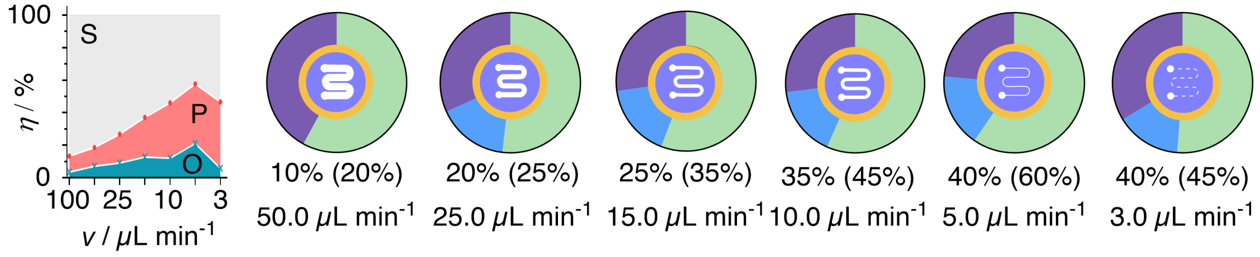

Figure S8. Interfacing organocatalyst **8** (0.2 equiv) with positive electric fields at constant current (*I* = 2.0 A) to facilitate monoterpene cyclization on multiwalled carbon nanotubes in electromicrofluidic reactor. Results are reported in Table S8. Substrate (grey), product (red) and unidentified products

(teal) plotted as a function of the flowrate and doughnut charts for product distribution with total yield (conversion) below each chart.

Table S8. Optimization of flowrate for monoterpene cyclizations catalyzed by positive electric fields in presence of catalyst **8** on multiwalled carbon nanotubes in electromicrofluidic reactors.[a]

Entry	v [μL·min^{-1}][b]	V [V][c]	η [%][d]	P [%][e]					O [%][f]	S [%][g]	P+O [%][h]
				**2**	**3**	**4**	**5**	**6**			
1	50	4.0	12	5	0	7	0	0	7	81	19
2	25	4.2	18	6	3	9	0	0	9	73	27
3	15	4.7	25	7	4	14	0	0	12	63	37
4	5	3.7[i]	38	9	6	23	0	0	20	42	58
5	3	3.9	42	14	6	22	0	0	5	53	47

[a] Conditions: Pt cathode, Gr-MWCNTs anode, I = 2.0 A, **8** (0.2 equiv), toluene. [b] Flowrate, in *micro*liters per minute. [c] Measured voltage, in volt, fluctuated within ± 300 mV range. [d] Total yield of main cyclic monoterpenes. [e] Yields of assigned cyclic products after one passage through the electromicrofluidic reactor. [f] Yields of other unidentified products, including loss of volatile materials and decomposition. [g] Substrate left after one passage through the electromicrofluidic reactor. [h] Total conversion. [i] Voltage fluctuation was ≈ 0. Conversions and yields were calculated from obtained GC traces. Summary of corresponding data presented in Figure S8.

6.4. Dependence on Temperature

The flow reactor was placed in cryo chamber to ensure 0 °C temperature. Then following a general procedure as described in Chapter 6.2, a stock solution of neryl chloride **1** (100 mM, 1.0 equiv) and *n*-decane (0.5 equiv) in anhydrous toluene (4.0 mL) and organocatalyst catalyst **8** (0.3 equiv) in anhydrous toluene (4.0 mL) were placed in separate syringes and pumped through T-mixer

into the electrochemical reactor at constant flowrate of 10 μL·min^{-1} to screen different current conditions at respective temperature (Figure S9). Conversions and yields were calculated from obtained GC traces by utilizing the Equations [S2]-[S6].

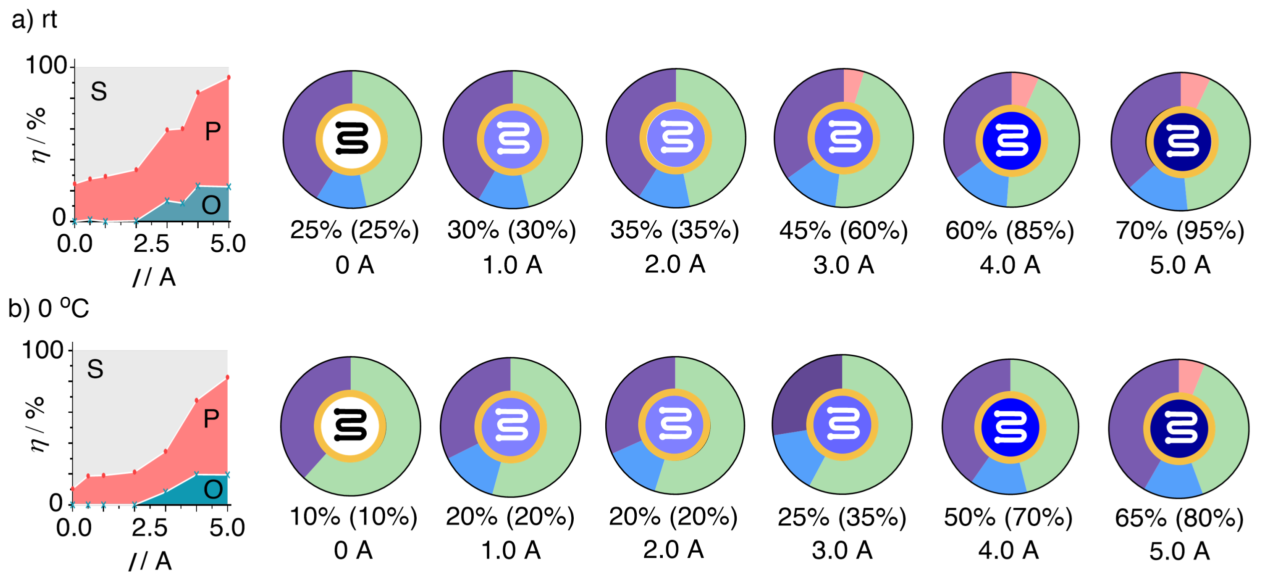

Figure S9. Optimization of temperature for monoterpene cyclization utilizing positive electric fields at constant flow (v = 10 μL·min^{-1}) in presence of organocatalyst **8** (0.3 equiv) to facilitate monoterpene cyclization on multiwalled carbon nanotubes in electromicrofluidic reactor at different temperatures: rt (a), 0 °C (b). Results are reported in Table S9. Substrate (grey), product (red) and unidentified products (teal) plotted as a function of the applied current and doughnut charts for product distribution with total yield (conversion) below each chart.

Table S9. Temperature optimization for monoterpene cyclizations catalyzed by positive electric fields in presence of catalyst **8** on multiwalled carbon nanotubes in electromicrofluidic reactors.[a]

Entry	I [A][b]	V [V][c]	η [%][d]	P [%][e]					O [%][f]	S [%][g]	P+O [%][h]
				**2**	**3**	**4**	**5**	**6**			
1	1.0	1.6	19	6	3	10	0	0	0	81	19
2	3.0	5.0[i]	26	7	4	15	0	0	8	66	34
3	4.0	4.7	48	19	7	22	0	0	20	32	68
4	5.0	4.6	63	26	9	24	0	4	20	17	83

[a] Conditions: Pt cathode, Gr-MWCNTs anode, **8** (0.3 equiv), 0 °C, toluene, 10 μL·min^{-1} flowrate. [b] Current applied, in ampere. [c] Measured voltage, in volt. [d] Total yield of main cyclic monoterpenes. [e] Yields of assigned cyclic products after one passage through the electromicrofluidic reactor. [f] Yields of other unidentified products, including loss of volatile materials and decomposition. [g] Substrate left after one passage through the electromicrofluidic reactor. [h] Total conversion. [i] Voltage fluctuated within ± 400 mV range. Conversions and yields were calculated from obtained GC traces. Summary of corresponding data presented in Figure S9.

6.5. Dependence on Base

The flow reactor was placed in cryo chamber to ensure 18 ºC temperature. Then following a general procedure as described in Chapter 6.2, solutions of neryl chloride **1** (100 mM, 1.0 equiv), 2,4,6-collidine or 2,6-di-*tert*-butylpyridine as a base (1.0 equiv) and *n*-decane (0.5 equiv) in anhydrous toluene (4.0 mL) and organocatalyst **8** (0.2 or 0.3 equiv, respectively) in anhydrous toluene (4.0 mL) were placed in separate syringes and pumped through T-mixer into the electrochemical reactor. Different current conditions at constant flowrate of 10 μL·min^{-1} (Figure S10a,b) and different flowrates at constant current I (4.0 A) (Figure S10c) were screened at 18 ºC. Conversions and yields were calculated from obtained GC traces by utilizing the Equations [S2]-[S6].

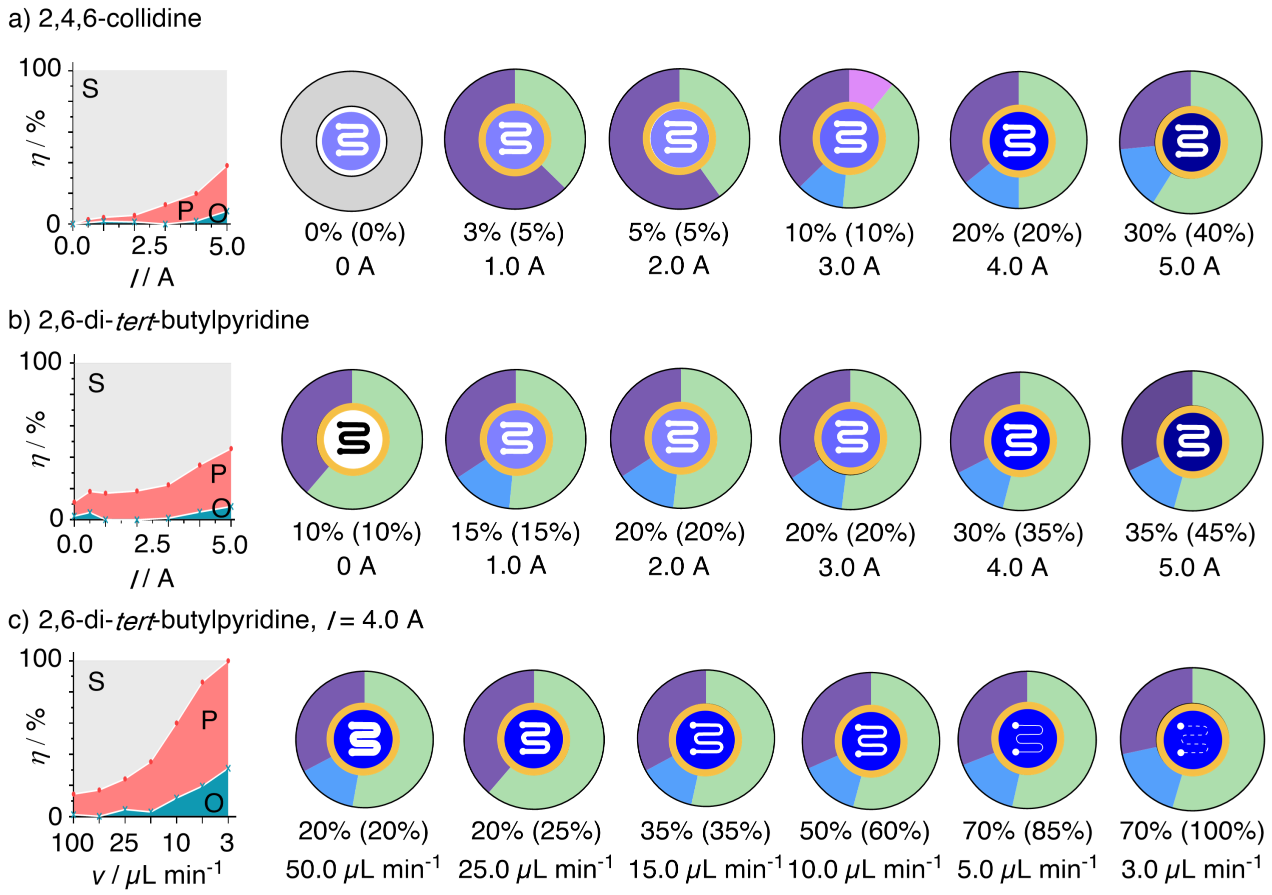

Figure S10. Interfacing organocatalyst **8** (0.2 equiv; (a), and 0.3 equiv; (b,c)) with positive electric fields at constant flow (v = 10 μL·min^{-1}) to facilitate monoterpene cyclization on multiwalled carbon nanotubes in electromicrofluidic reactor while screening different bases: 2,4,6-collidine (a), 2,6-di-*tert*-butylpyridine (b); and different flowrates at I = 4.0 A in presence of 2,6-di-*tert*-butylpyridine as a base (c). Results (b,c) are reported in Tables S10 and S11. Substrate (grey), product (red) and unidentified products (teal) plotted as a function of the applied current (or flowrate in (c) case) and doughnut charts for product distribution with total yield (conversion) below each chart.

Table S10. Monoterpene cyclizations catalyzed by positive electric fields in presence of catalyst **8** and base on multiwalled carbon nanotubes in electromicrofluidic reactors.[a]

Entry	I [A][b]	V [V][c]	η [%][d]	P [%][e]					O [%][f]	S [%][g]	P+O [%][h]
				**2**	**3**	**4**	**5**	**6**			
1	0	0.6	9	4	0	5	0	0	2	89	11
2	1.0	1.2	17	6	2	9	0	0	0	83	17
3	2.0	2.3	18	6	2	10	0	0	0	82	18
4	3.0	3.5	21	7	3	11	0	0	1	78	22
5	4.0	4.3	30	10	4	16	0	0	5	65	35
6	5.0	4.0	37	12	5	20	0	0	9	54	46

[a] Conditions: Pt cathode, Gr-MWCNTs anode, **8** (0.3 equiv), 2,6-di-*tert*-butylpyridine (1.0 equiv), 18 °C, 10 μL·min^{-1} flowrate, toluene. [b] Current applied, in ampere. [c] Measured voltage, in volt. [d] Total yield of main cyclic monoterpenes. [e] Yields of assigned cyclic products after one passage through the electromicrofluidic reactor. [f] Yields of other unidentified products, including loss of volatile materials and decomposition. [g] Substrate left after one passage through the electromicrofluidic reactor. [h] Total conversion. Conversions and yields were calculated from obtained GC traces. Summary of corresponding data presented in Figure S10b.

Table S11. Flowrate optimization for monoterpene cyclizations catalyzed by positive electric fields in presence of catalyst **8** and base on multiwalled carbon nanotubes in electromicrofluidic reactors.[a]

Entry	v [µL·min^{-1}][b]	V [V][c]	η [%][d]	P [%][e]					O [%][f]	S [%][g]	P+O [%][h]
				**2**	**3**	**4**	**5**	**6**			
1	100	3.6	13	4	2	7	0	0	1	86	14
2	50	3.8	17	6	2	9	0	0	0	83	17
3	25	4.0	20	8	0	12	0	0	4	76	24
4	15	4.4	33	11	4	18	0	0	2	65	35
5	10	4.6	50	16	7	27	0	0	10	40	60
6	5.0	4.6	69	21	11	37	0	0	17	14	86
7	3.0	4.4	71	20	12	39	0	0	29	0	100

[a] Conditions: Pt cathode, Gr-MWCNTs anode, I = 4.0 A, **8** (0.3 equiv), 2,6-di-*tert*-butylpyridine (1.0 equiv), 18 °C, toluene. [b] Flowrate, in *micro*liters per minute. [c] Measured voltage, in volt. [d] Total yield of main cyclic monoterpenes. [e] Yields of assigned cyclic products after one passage through the electromicrofluidic reactor. [f] Yields of other unidentified products, including loss of volatile materials and decomposition. [g] Substrate left after one passage through the electromicrofluidic reactor. [h] Total conversion. Conversions and yields were calculated from obtained GC traces. Summary of corresponding data presented in Figure S10c.

7. Hydroquinone Oxidation Control

The experiment in flow was carried out in an undivided cell using a Vapourtec Ion Electrochemical Reactor (FEP spacer = 0.25 mm, reactor volume = 0.30 mL), employing a platinum electrode as the cathode and graphite electrode coated with MWCNTs as the anode and *vice versa* (active surface area = 12 cm^2 for each electrode). A solution of hydroquinone **9** (100 mM) in

acetonitrile was pumped into the electrochemical reactor with a 15 μL·min^{-1} flowrate, and increasing current values were screened. The first one and a half reactor volumes (= 0.45 mL) were disposed to ensure that a steady state of the system had been reached. After collection for 10 min, the reaction mixture was diluted for *ca* 200 times and analyzed by UV-vis spectrometry.

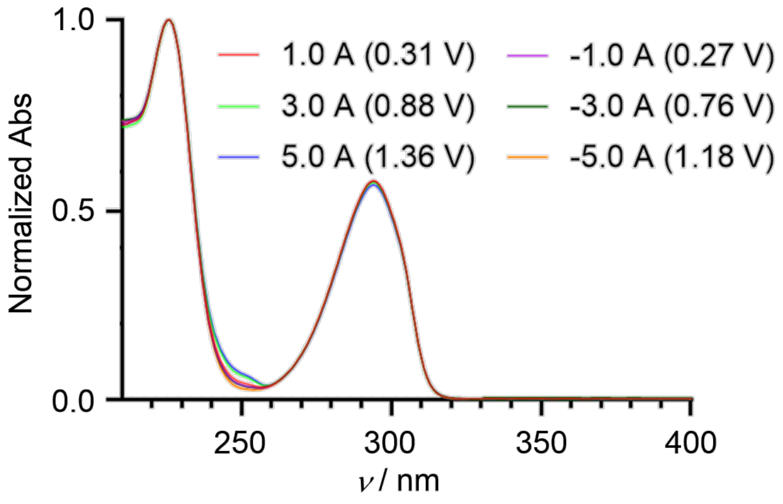

Figure S11. Normalized UV-vis spectra of hydroquinone **9** and the reaction mixtures. The concentration is approximately 0.5 mM.

8. GC Traces

8.1. Reference Compounds

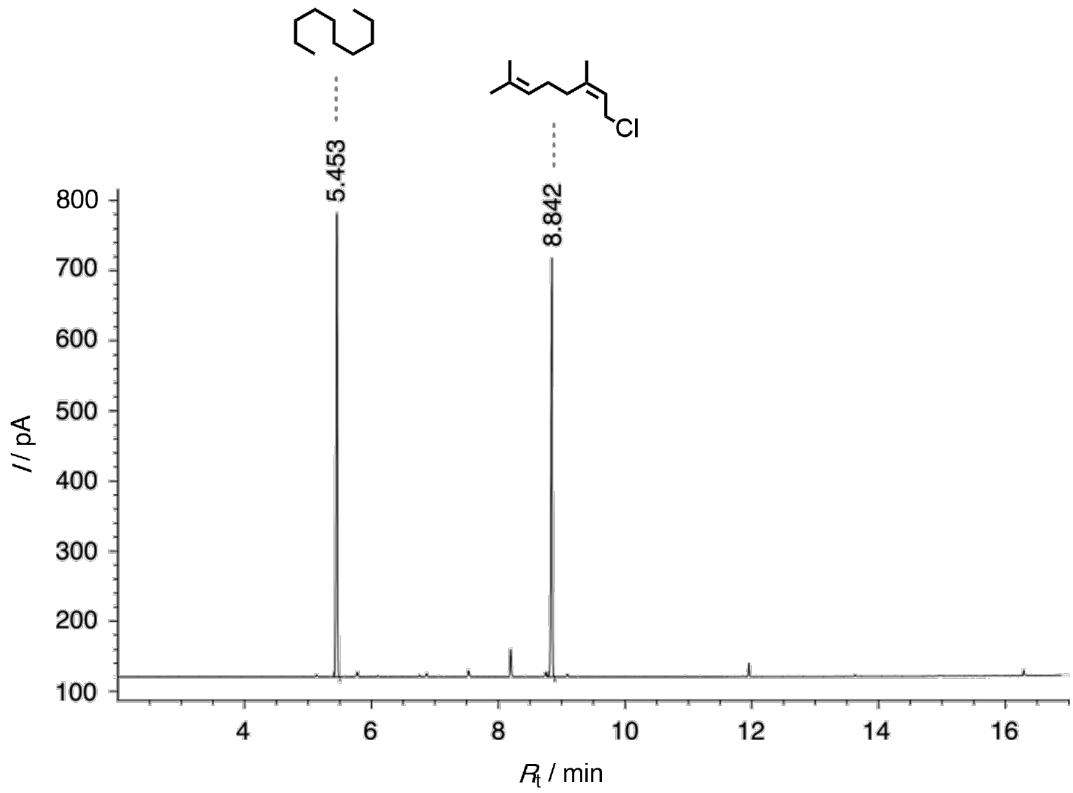

Figure S12. GC trace of neryl chloride **1** and *n*-decane used as an internal standard. Synthesized neryl chloride used in this study contained a small (4-7%) impurity of α-terpineol depending on the batch.

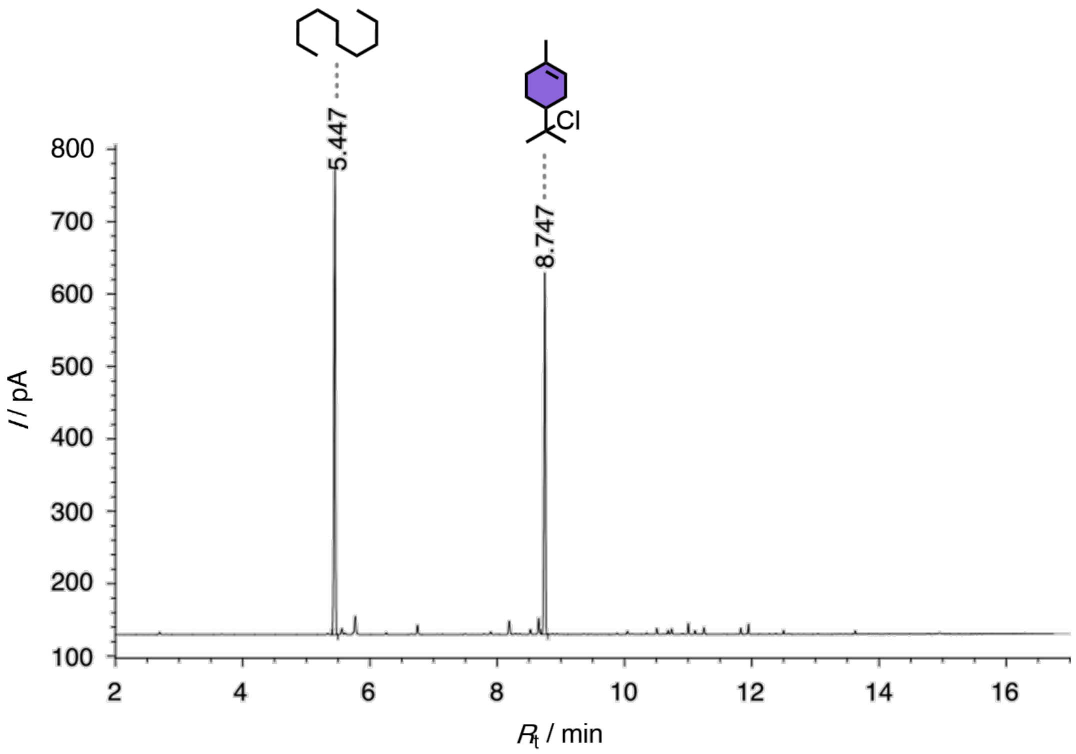

Figure S13. GC trace of α-terpinyl chloride **2** and *n*-decane used as an internal standard.

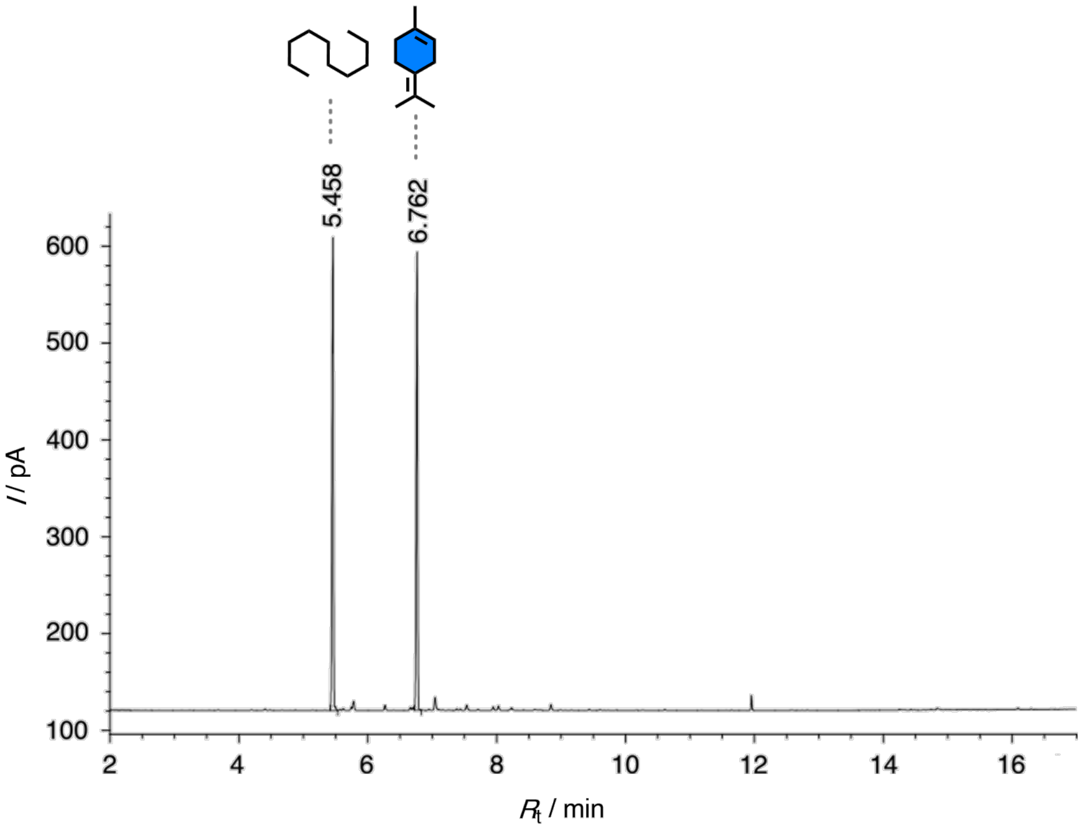

Figure S14. GC trace of terpinolene **3** and *n*-decane used as an internal standard.

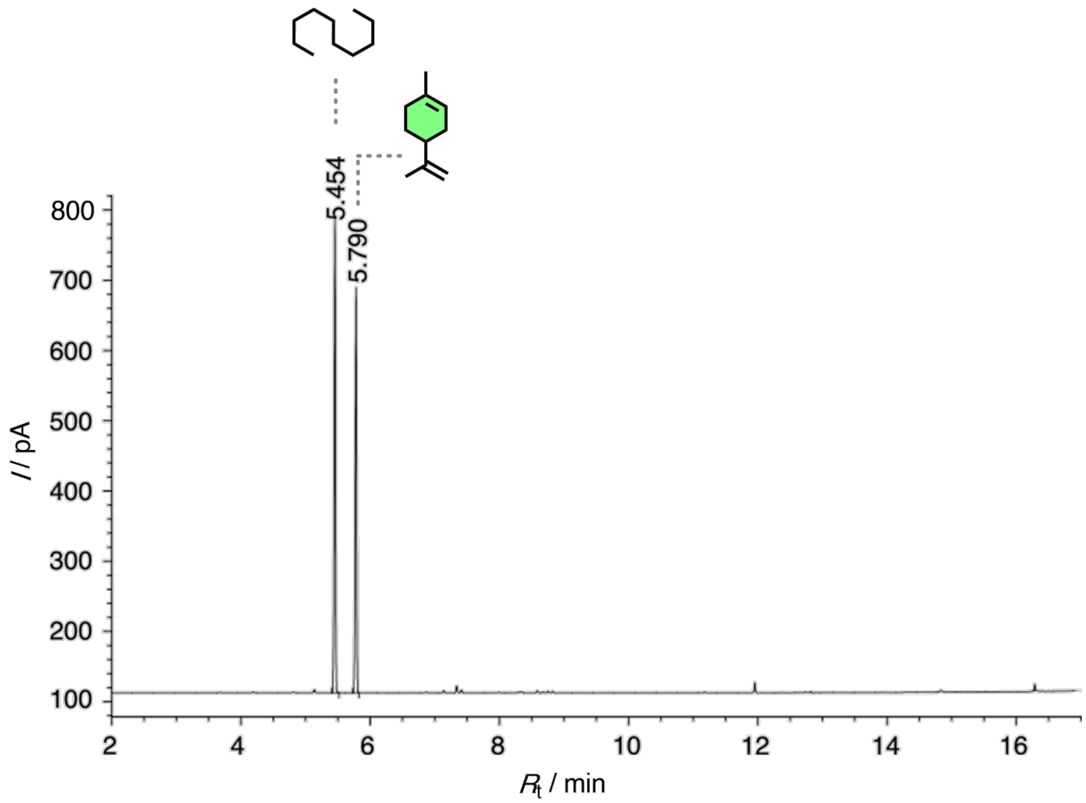

Figure S15. GC trace of limonene **4** and *n*-decane used as an internal standard.

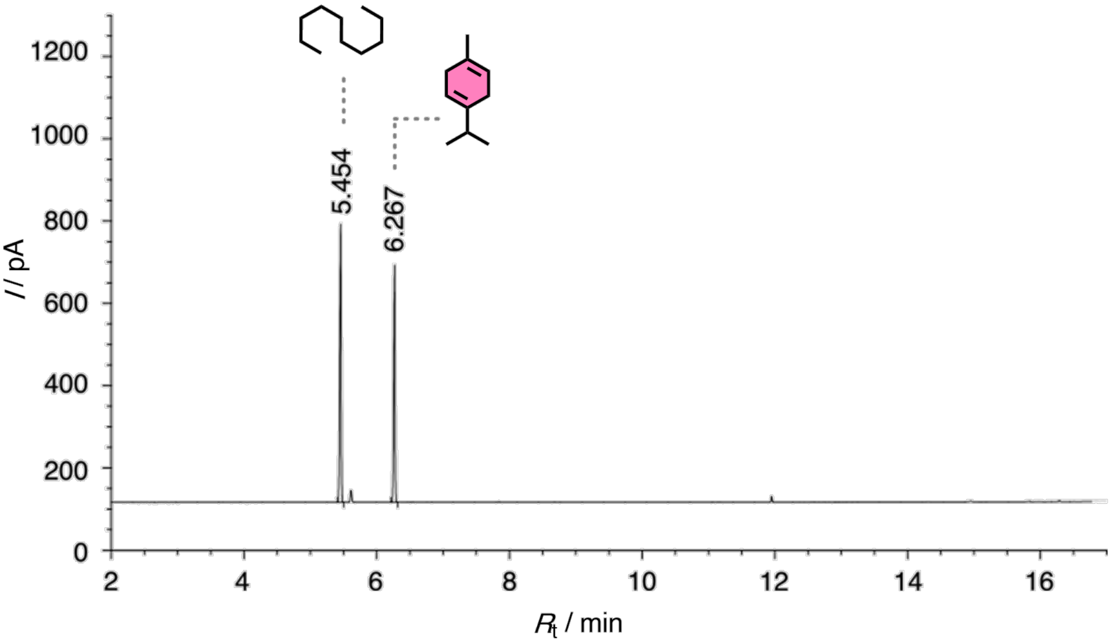

Figure S16. GC trace of γ-terpinene **5** and *n*-decane used as an internal standard.

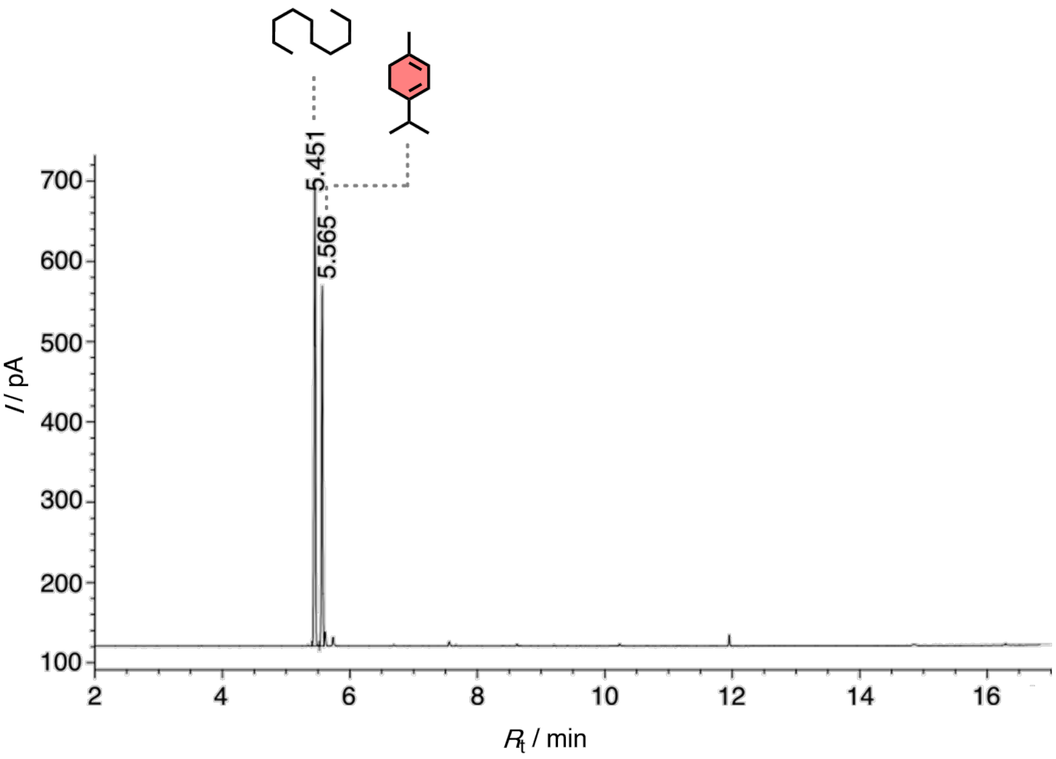

Figure S17. GC trace of α-terpinene **6** and *n*-decane used as an internal standard.

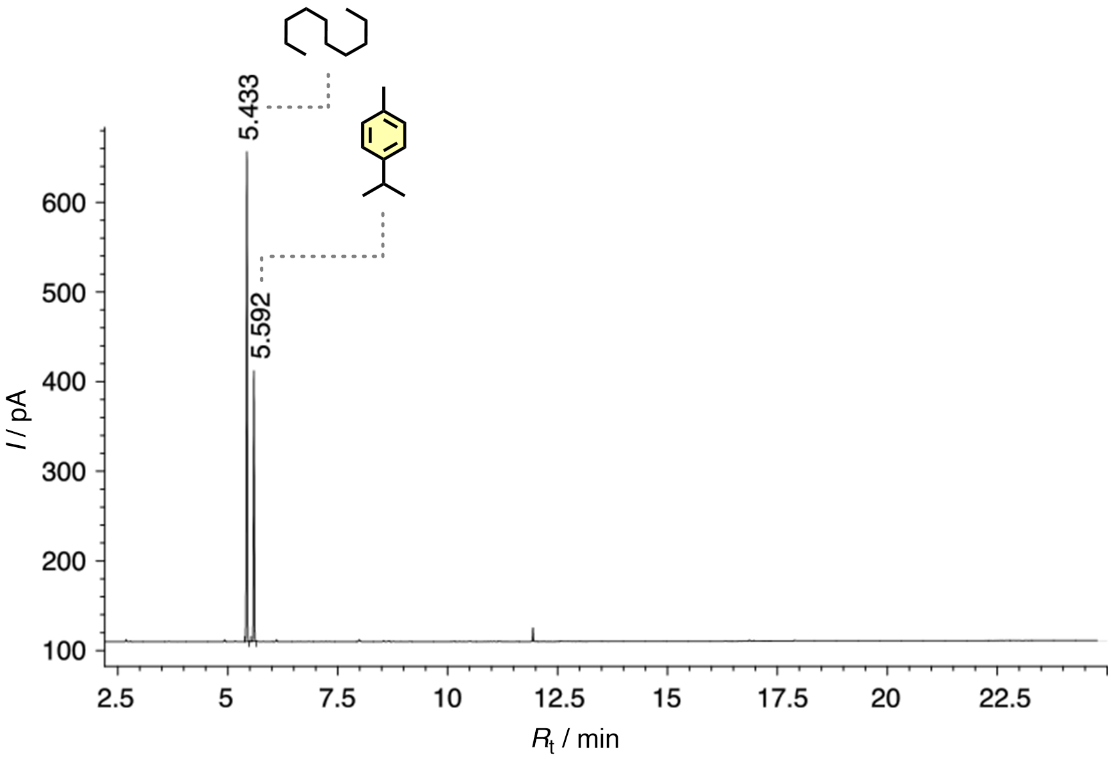

Figure S18. GC trace of p-cymene **7** and *n*-decane used as an internal standard.

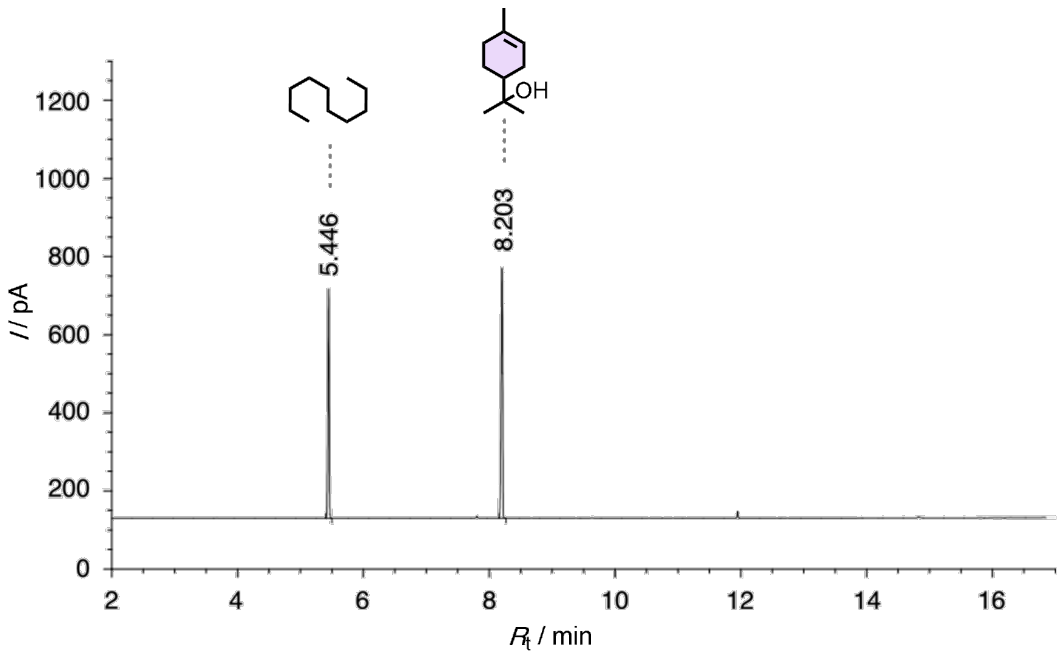

Figure S19. GC trace of α-terpineol and *n*-decane used as an internal standard.

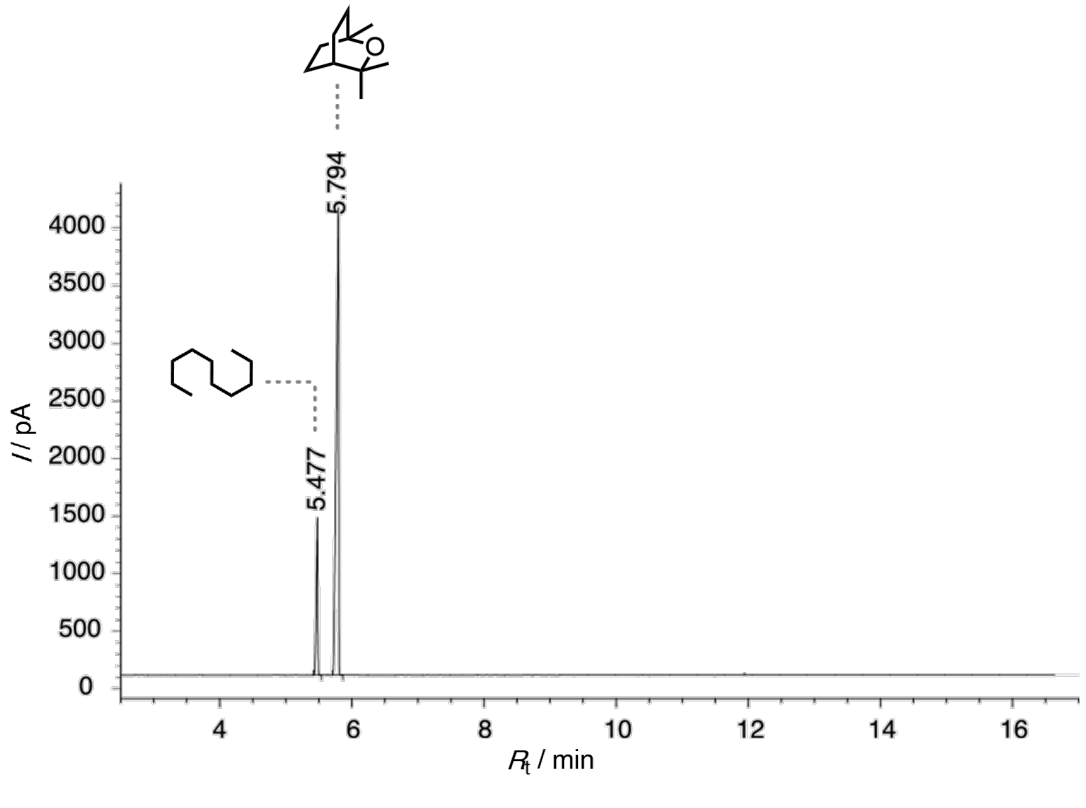

Figure S20. GC trace of eucalyptol and *n*-decane used as an internal standard.

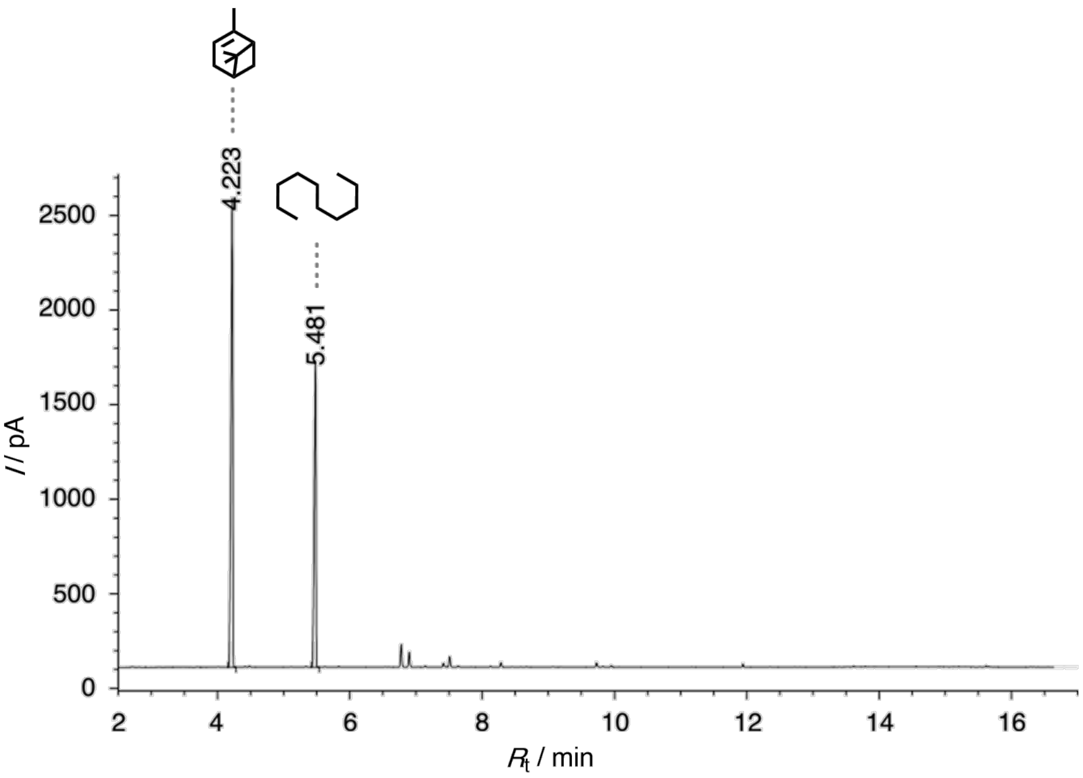

Figure S21. GC trace of α-pinene and *n*-decane used as an internal standard.

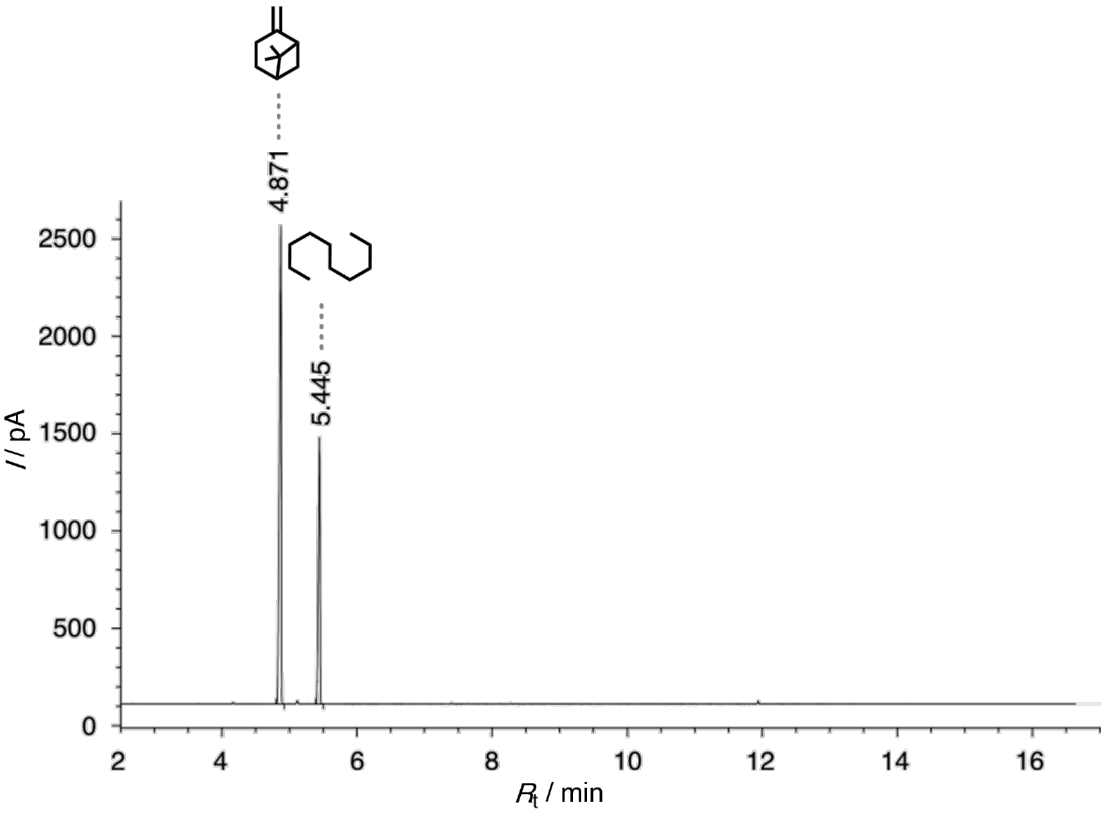

Figure S22. GC trace of γ-pinene and *n*-decane used as an internal standard.

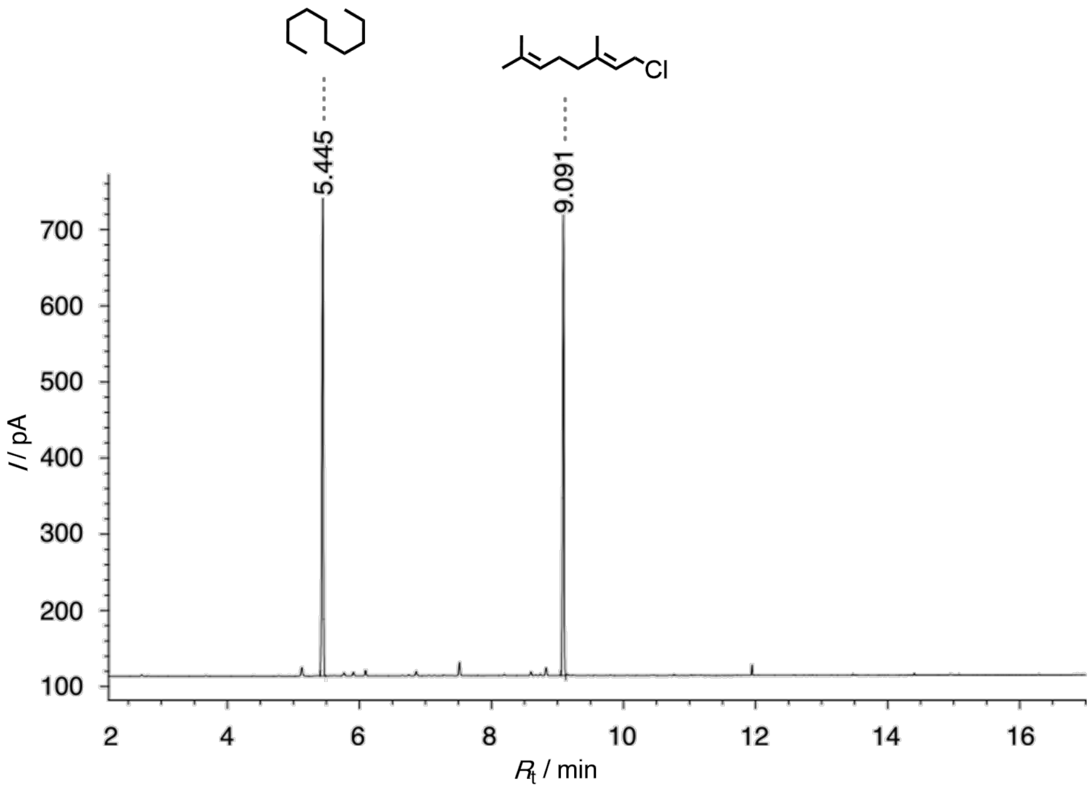

Figure S23. GC trace of geranyl chloride and *n*-decane used as an internal standard.

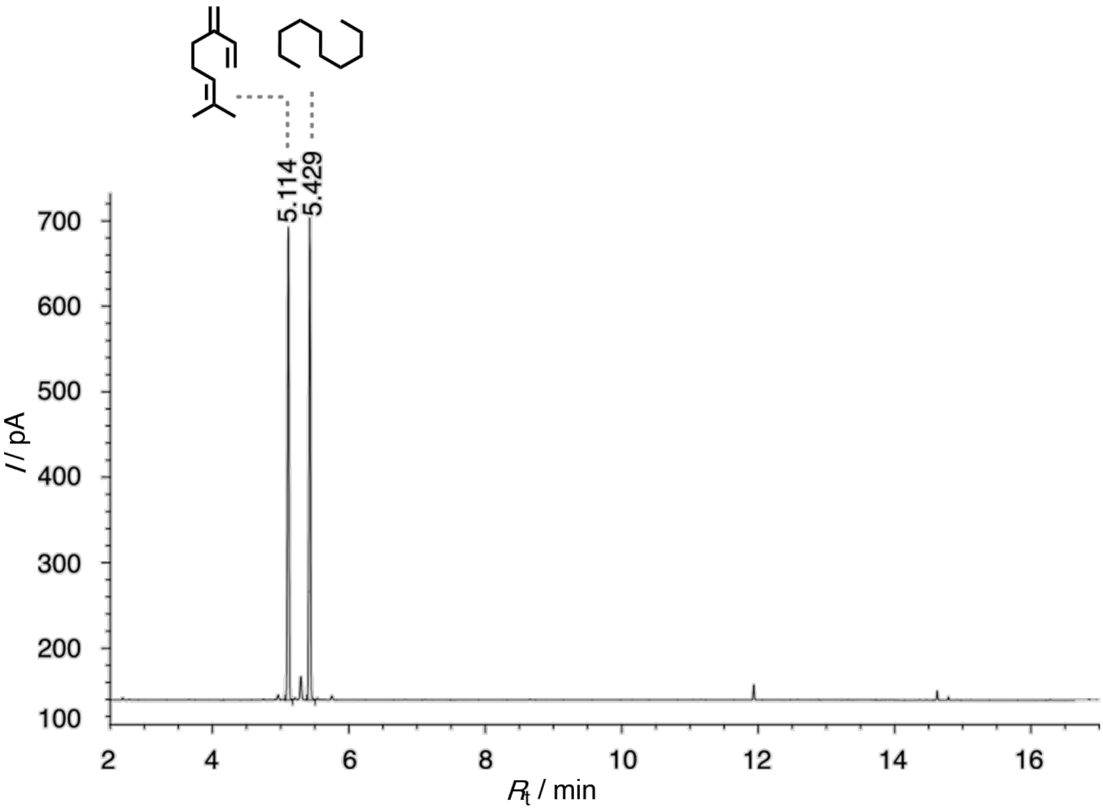

Figure S24. GC trace of myrcene and *n*-decane used as an internal standard.

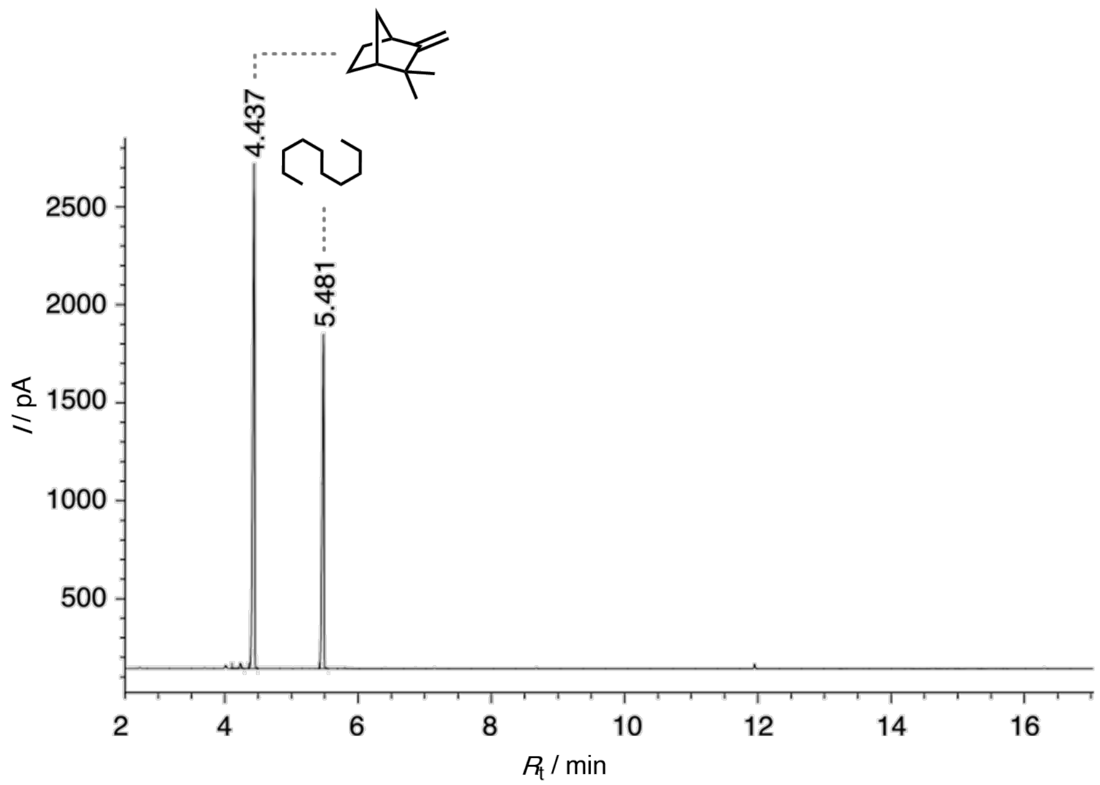

Figure S25. GC trace of camphene and *n*-decane used as an internal standard.

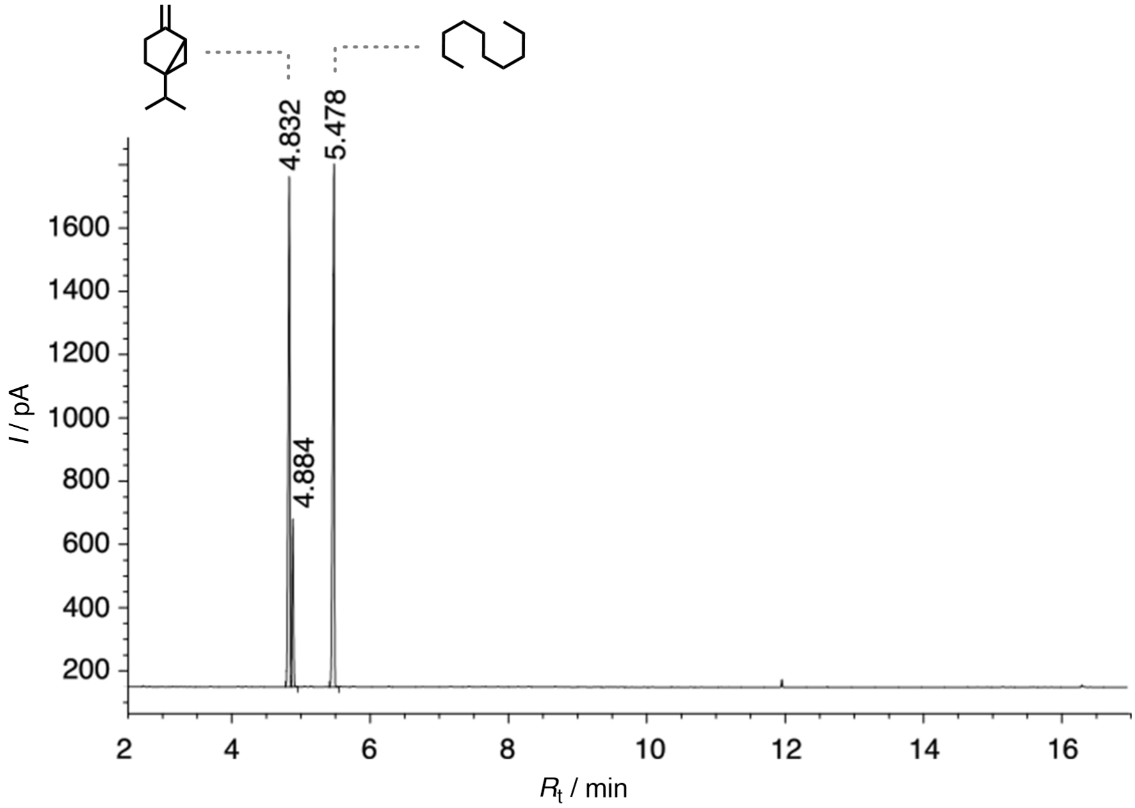

Figure S26. GC trace of sabinene and *n*-decane used as an internal standard.

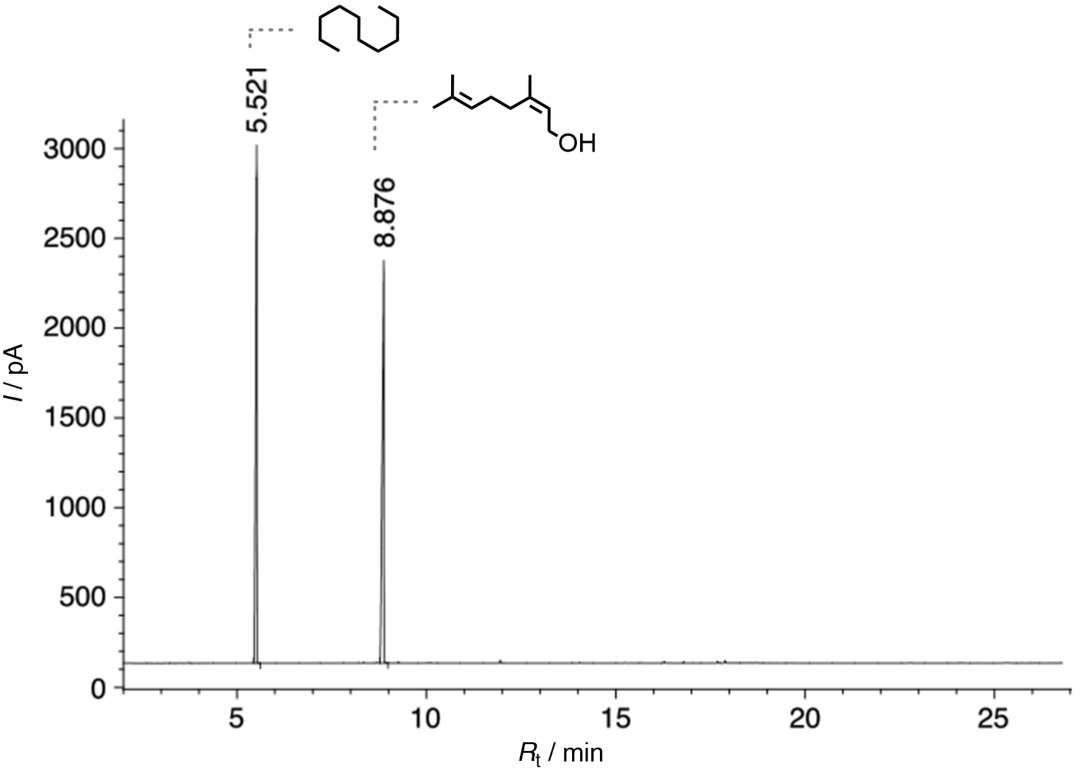

Figure S27. GC trace of nerol **11** and *n*-decane used as an internal standard.

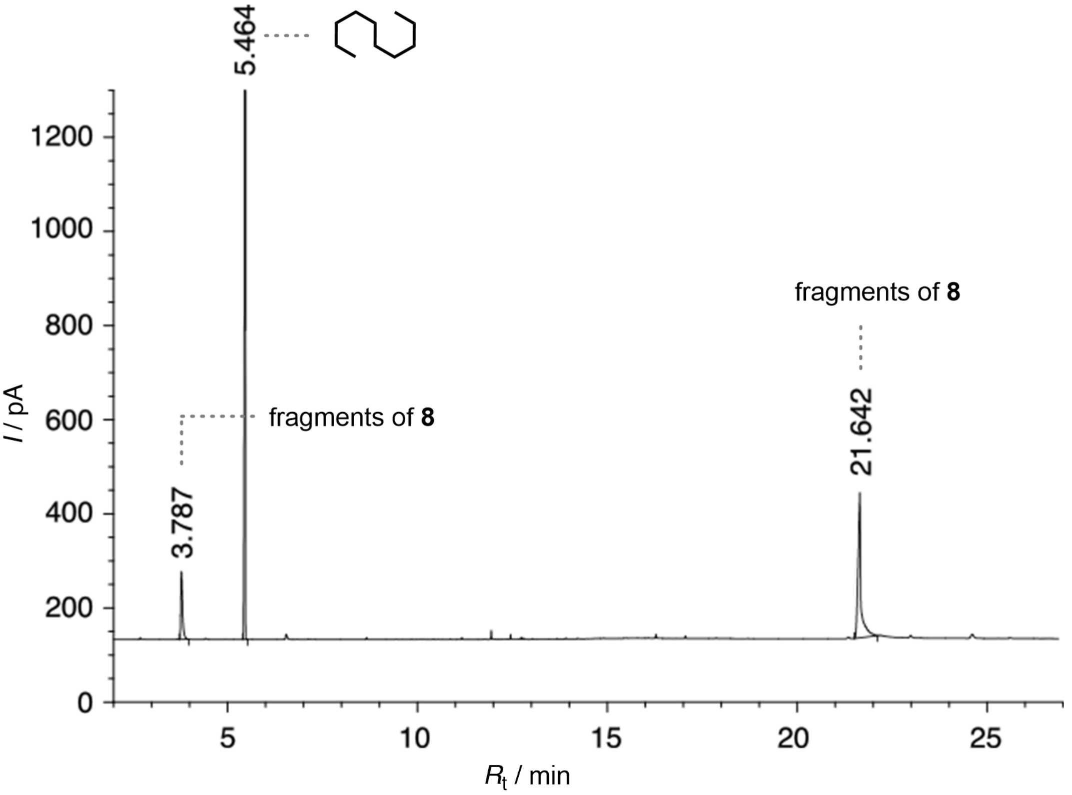

Figure S28. GC trace of organocatalyst **8** and *n*-decane used as an internal standard. *Note:* Two peaks attributed to catalyst were observed presumably due to organocatalyst decomposition during injection at high temperatures.

8.2. Reaction Mixtures

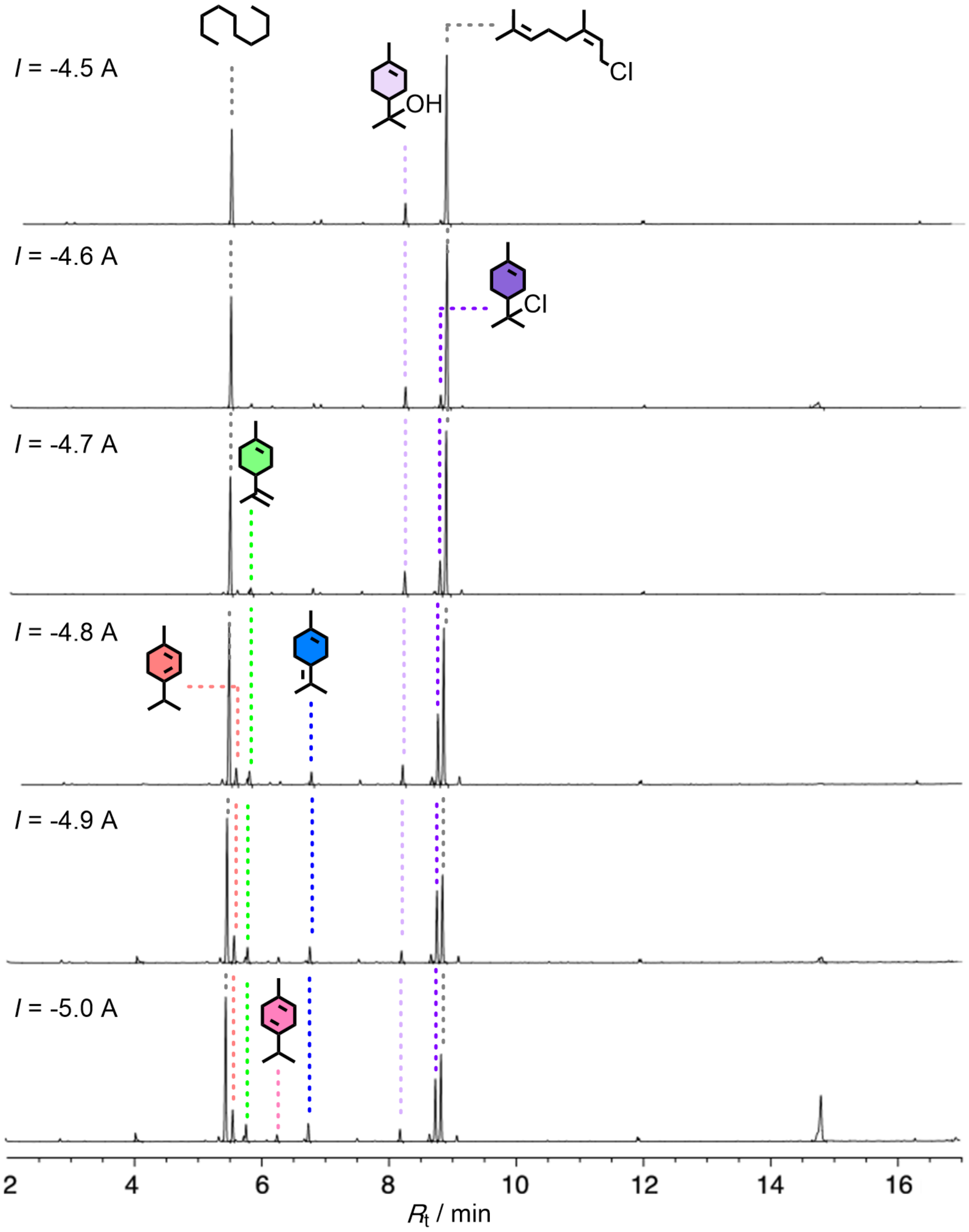

Figure S29. GC trace of neryl chloride **1** cyclization in toluene catalyzed by negative electric fields at constant flow (v = 15 μL·min^{-1}) on multiwalled carbon nanotubes in electromicrofluidic reactor, results are reported in Table S2 and Figures 2b, c, S3. Neryl chloride used in this study contained a small (8%) impurity of α-terpineol.

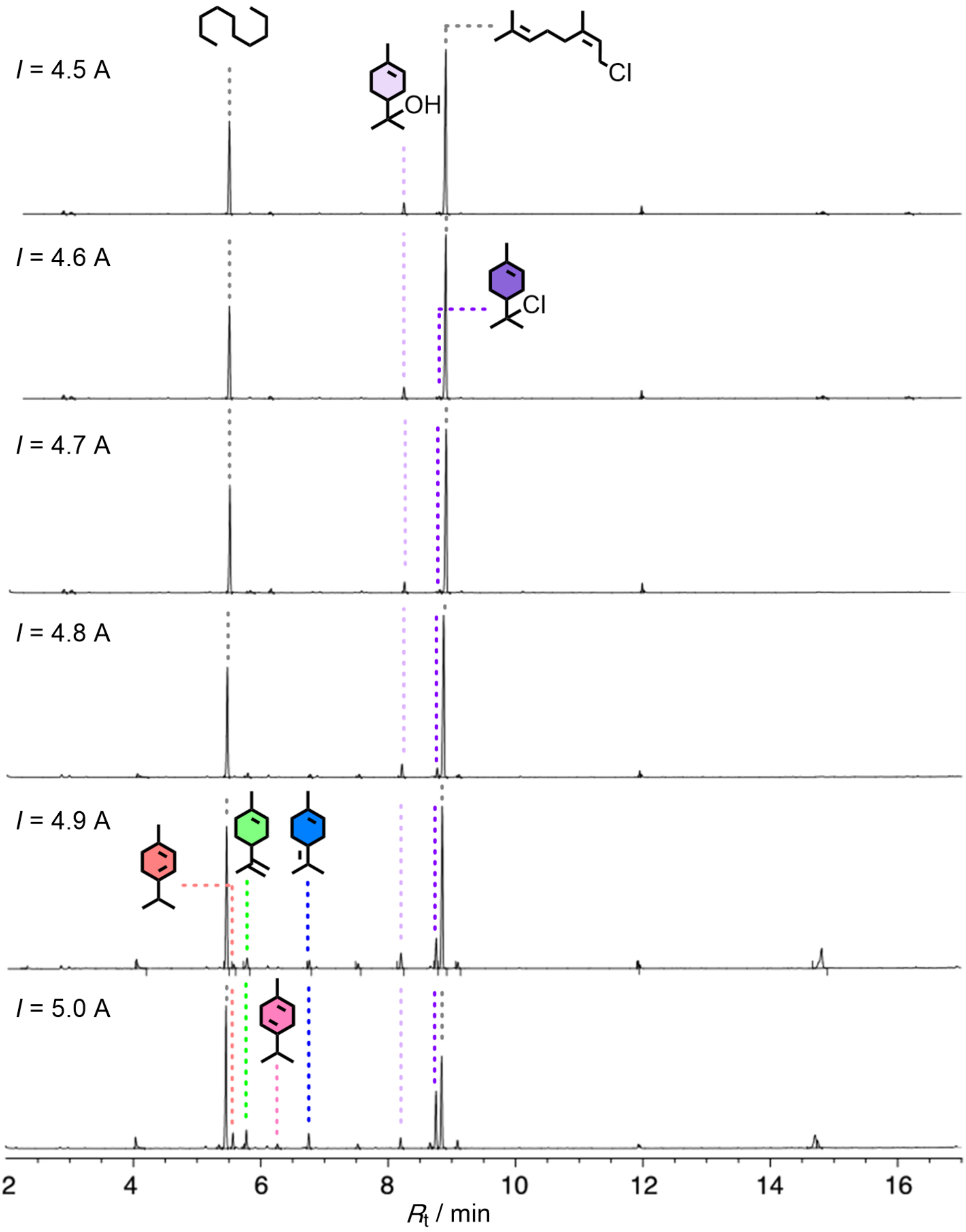

Figure S30. GC trace of neryl chloride **1** cyclization in toluene catalyzed by positive electric fields at constant flow (v = 15 µL·min^{-1}) on multiwalled carbon nanotubes in electromicrofluidic reactor, results are reported in Table S3 and Figures 2c, d, S3. Neryl chloride used in this study contained a small (5%) impurity of α-terpineol.

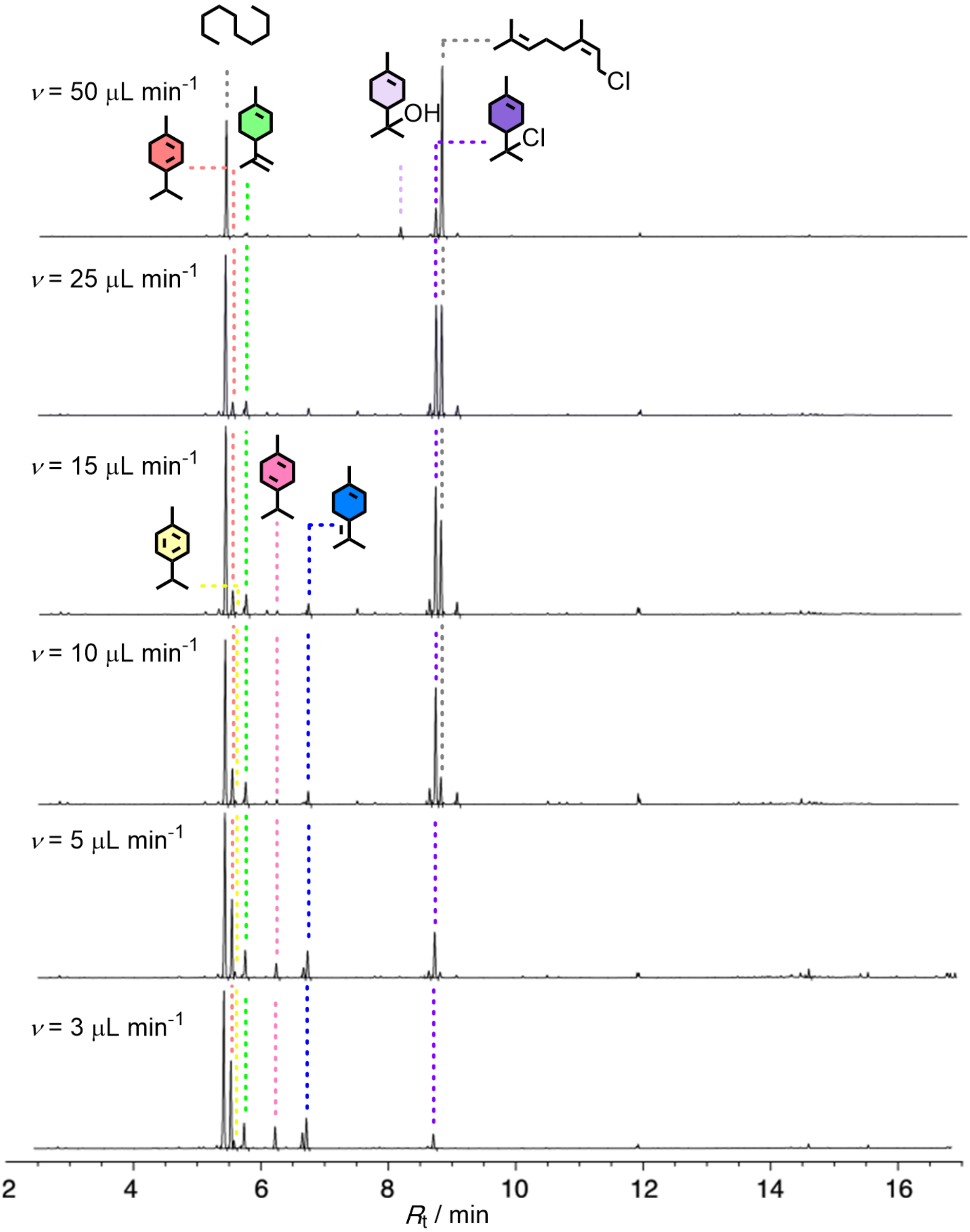

Figure S31. GC trace of neryl chloride **1** cyclization in toluene catalyzed by negative electric fields at current (I = 5.0 A) on multiwalled carbon nanotubes in electromicrofluidic reactor, results are reported in Table S4 and Figures 2e,f, S4. Neryl chloride used in this study contained a small (4%) impurity of α-terpineol.

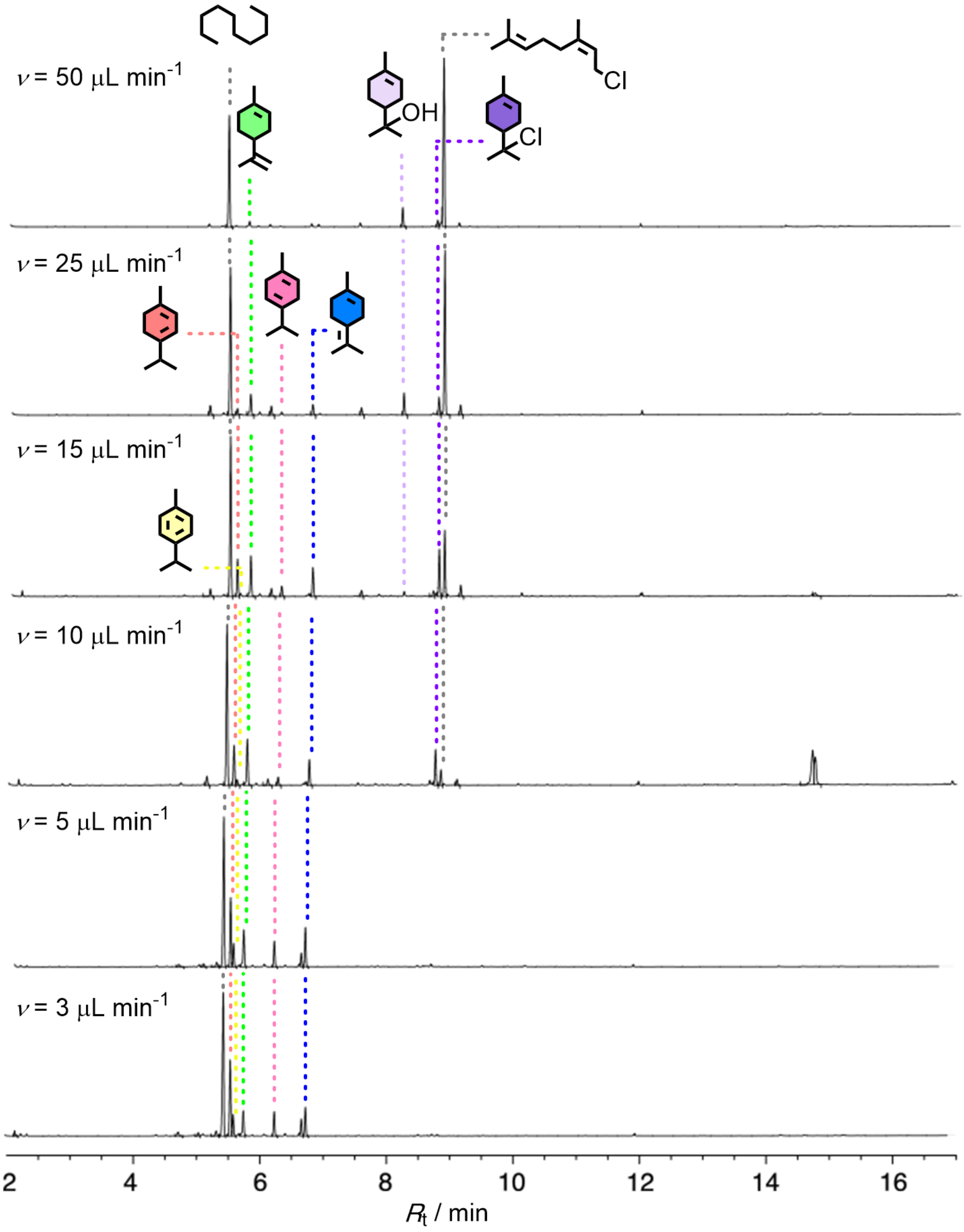

Figure S32. GC trace of neryl chloride **1** cyclization in toluene catalyzed by positive electric fields at constant current (I = 5.0 A) on multiwalled carbon nanotubes in electromicrofluidic reactor, results are reported in Table S5 and Figures 2g, h, S4. Neryl chloride used in this study contained a small (7%) impurity of α-terpineol.

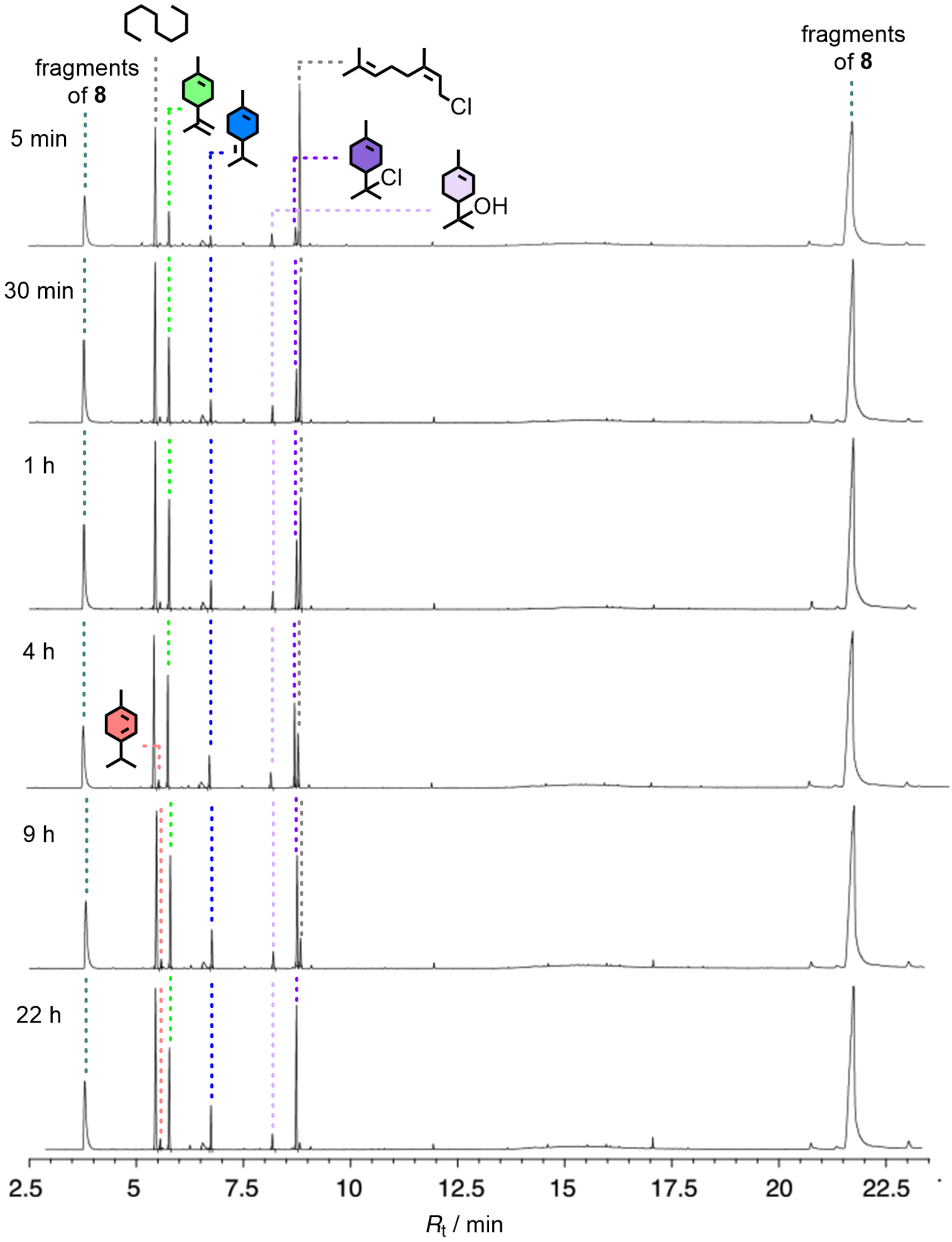

Figure S33. GC trace of neryl chloride **1** cyclization in presence of organocatalyst **8** (1.0 equiv) in bulk solution without electromicrofluidics, results are reported in Table S6 and Figures 3a, S5. Neryl chloride used in this study contained a small (7%) impurity of α-terpineol. *Note:* Intensity of catalyst's fragment peaks decreases with increasing field presumably due to HCl complexes[S2] interfering with GC fragmentation and detection.

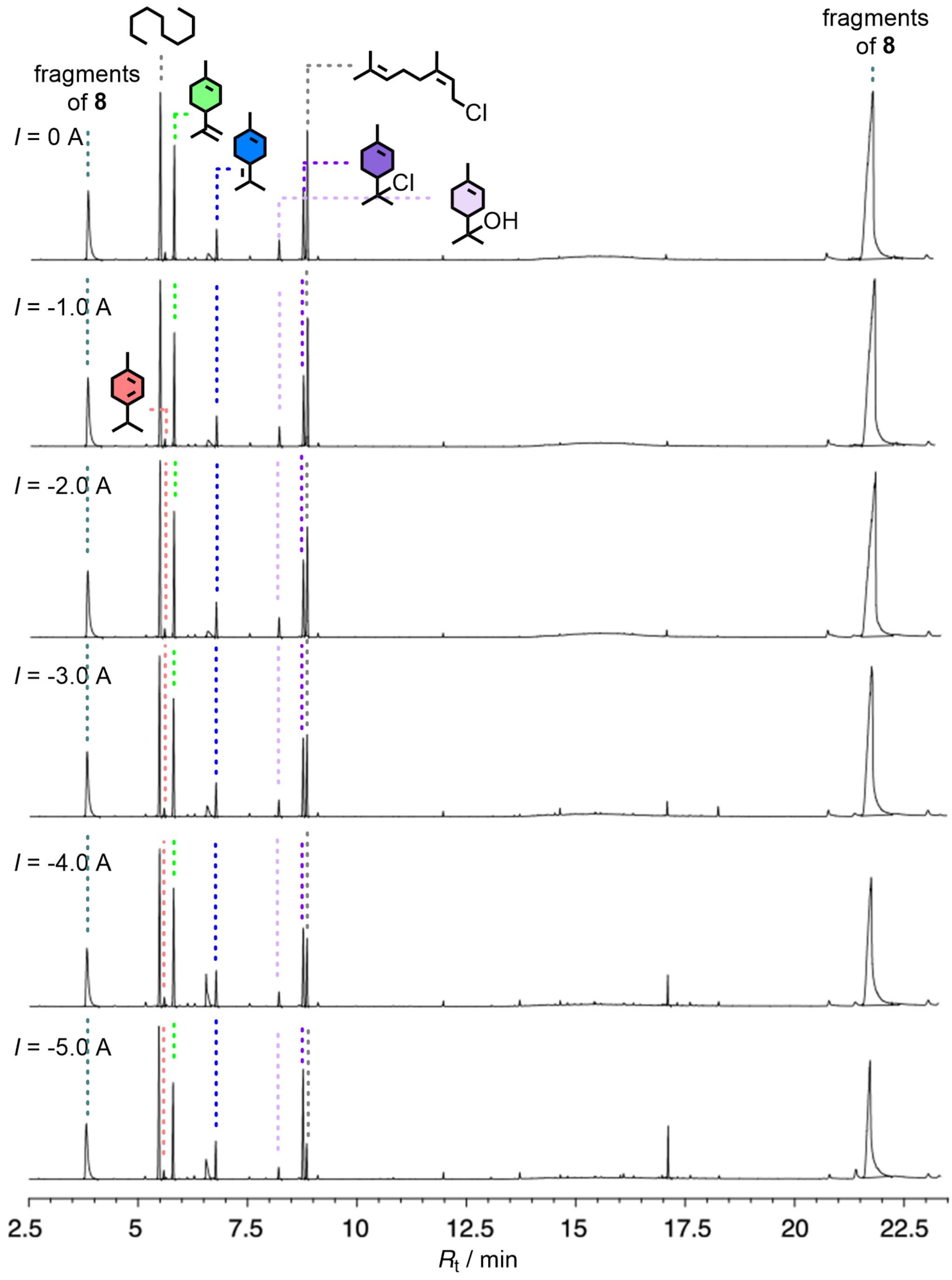

Figure S34. GC trace of neryl chloride **1** cyclization in toluene catalyzed by negative electric fields at constant flow (v = 10 μL·min^{-1}) in presence of 1.0 equiv of organocatalyst **8** on multiwalled carbon nanotubes in electromicrofluidic reactor, results are reported in Table S7 and Figures 3b, c, S6b. Neryl chloride used in this study contained a small (8%) impurity of α-terpineol. *Note:* Intensity of catalyst's fragment peaks decreases with increasing field presumably due to HCl complexes[S2] interfering with GC fragmentation and detection.

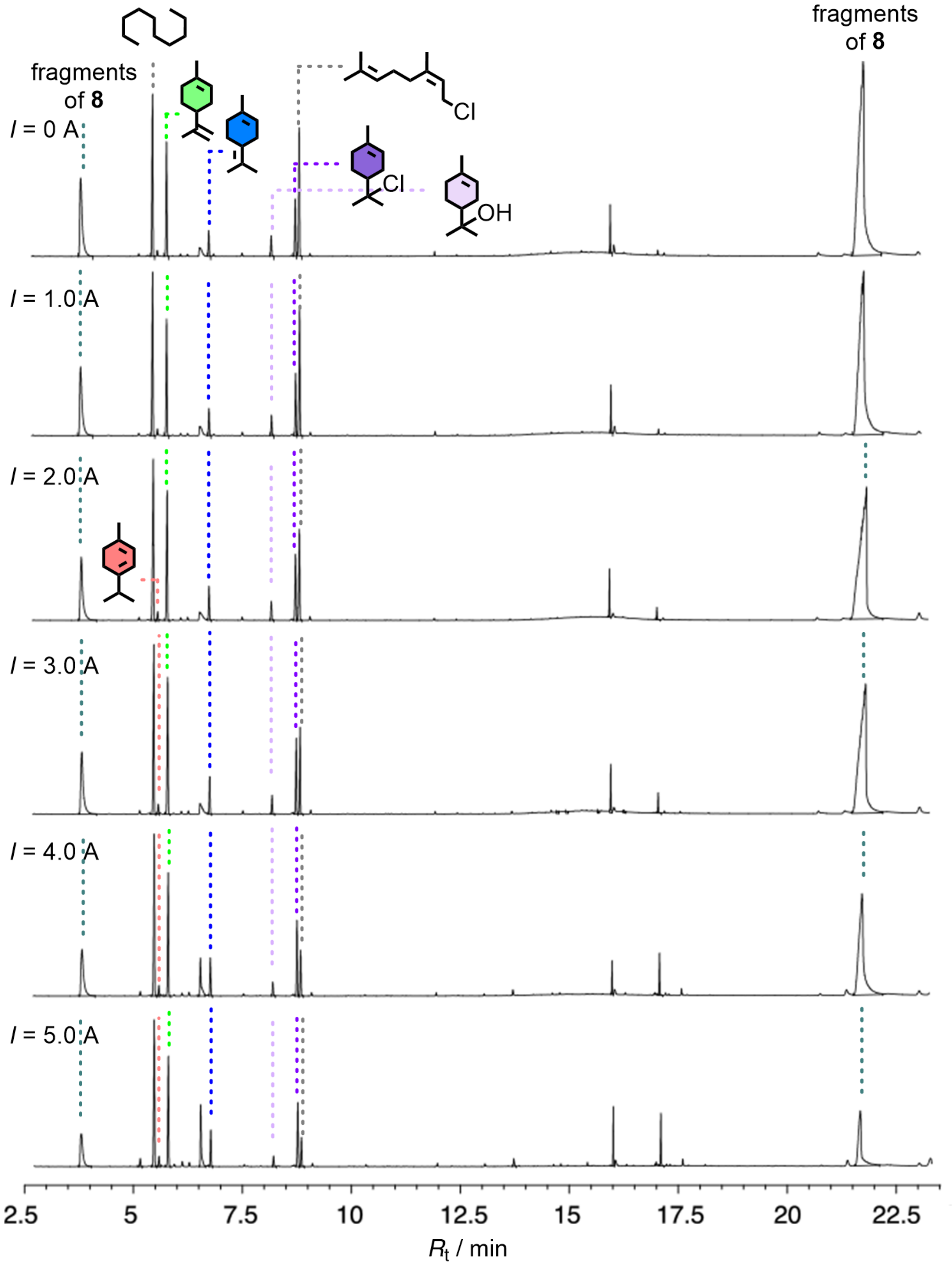

Figure S35. GC trace of neryl chloride **1** cyclization in toluene catalyzed by positive electric fields at constant flow ($v = 10$ µL·min^{-1}) in presence of 1.0 equiv of organocatalyst **8** on multiwalled carbon nanotubes in electromicrofluidic reactor, results are reported in Table S7 and Figures 3d, e, S7e. Neryl chloride used in this study contained a small (8%) impurity of α-terpineol. *Note:* Intensity of catalyst's fragment peaks decreases with increasing field presumably due to HCl complexes[S2] interfering with GC fragmentation and detection.

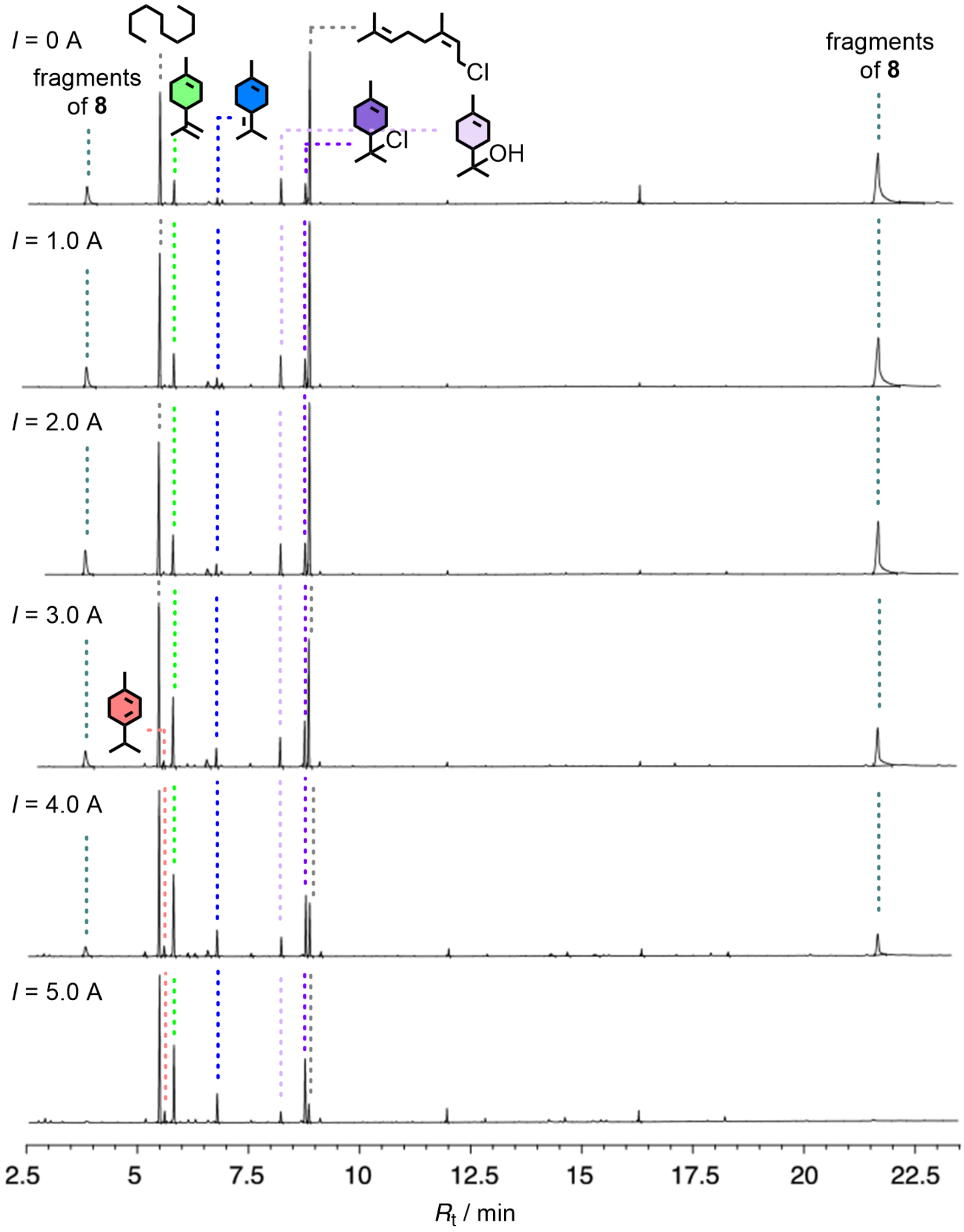

Figure S36. GC trace of neryl chloride **1** cyclization in toluene catalyzed by positive electric fields at constant flow (v = 10 μL·min^{-1}) in presence of 0.3 equiv of organocatalyst **8** on multiwalled carbon nanotubes in electromicrofluidic reactor, results are reported in Table S7 and Figures 3f, g, S6c. Neryl chloride used in this study contained a small (8%) impurity of α-terpineol. *Note:* Intensity of catalyst's fragment peaks decreases with increasing field presumably due to HCl complexes[S2] interfering with GC fragmentation and detection.

9. Supporting References

[S1] M. Ángeles Gutiérrez López, R. Ali, M.-L. Tan, N. Sakai, T. Wirth, S. Matile, *Sci. Adv.* **2023**, *9*, eadj5502.

[S2] D. A. Kutateladze, D. A. Strassfeld, E. N. Jacobsen, *J. Am. Chem. Soc.* **2020**, *142*, 6951–6956.

[S3] G. Gabriel, R. Gómez-Martínez, R. Villa, *Physiol. Meas.* **2008**, *29*, 203–212.

[S4] Q. Zhang, K. Tiefenbacher, *Nat. Chem.* **2015**, *7*, 197–202.

The original data can be found at: https://doi.org/10.5281/zenodo.13165225.

10. NMR Spectra

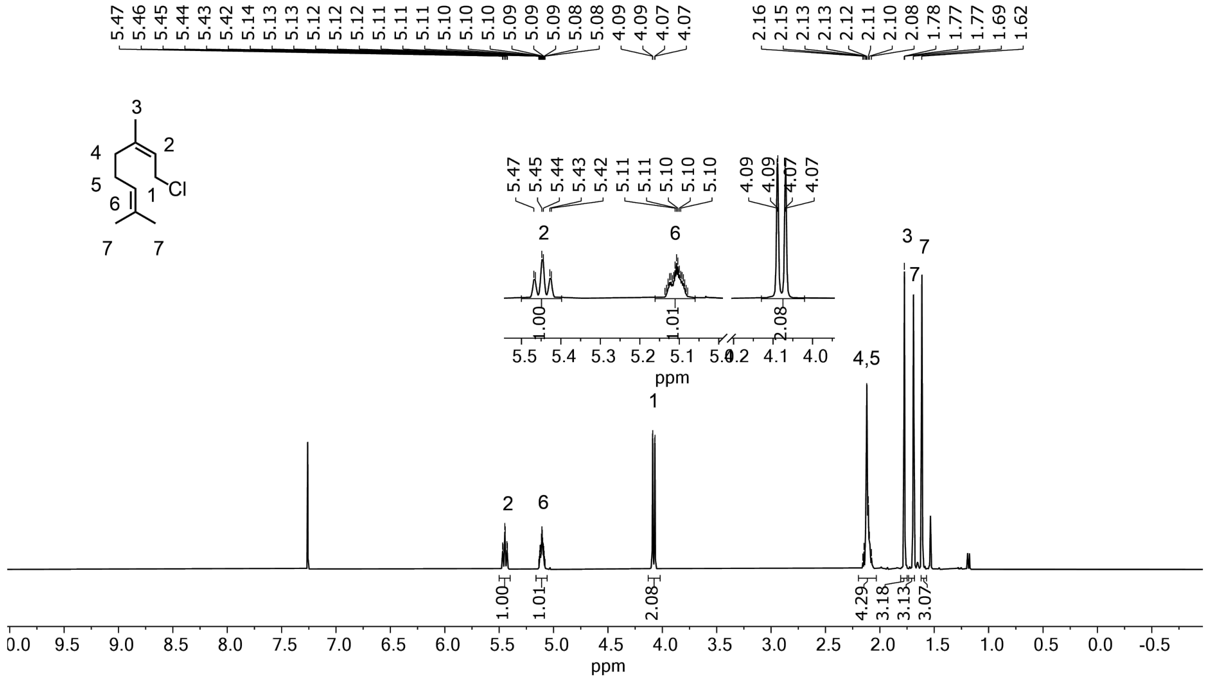

Figure S37. 400 MHz ^{1}H NMR spectrum ($CDCl_3$) of compound **1.**

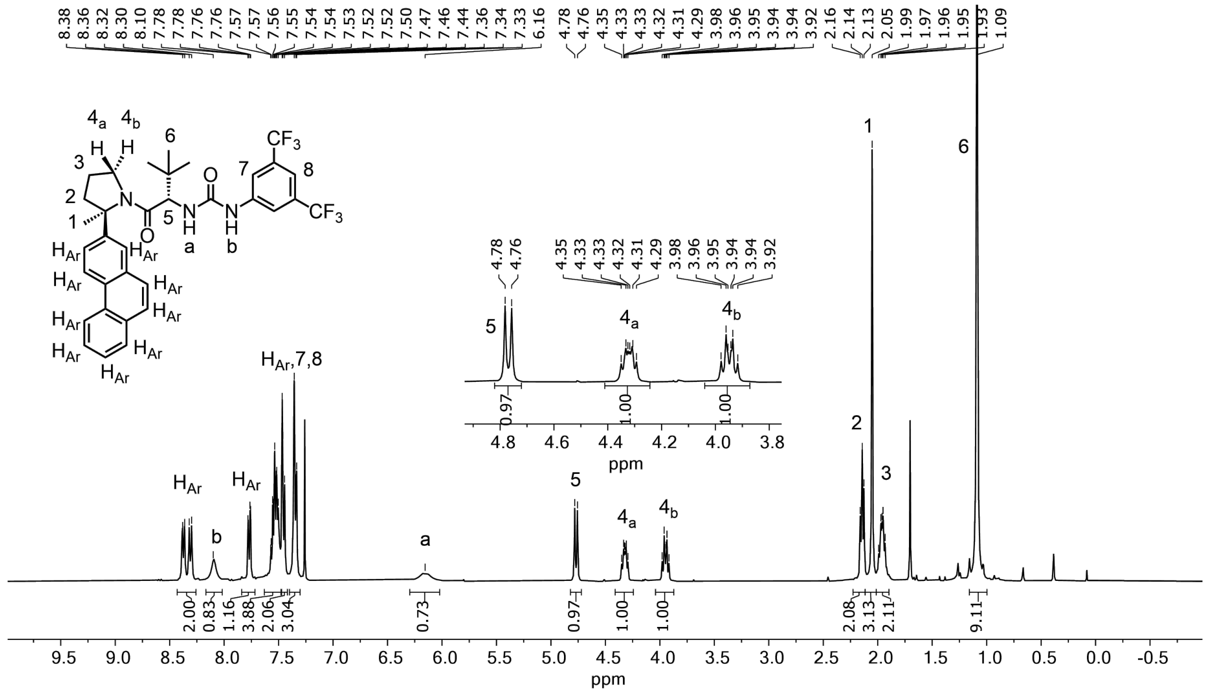

Figure S38. 400 MHz ^{1}H NMR spectrum ($CDCl_3$) of organocatalyst **8** is in accordance to reported ^{1}H NMR spectrum.[S2]

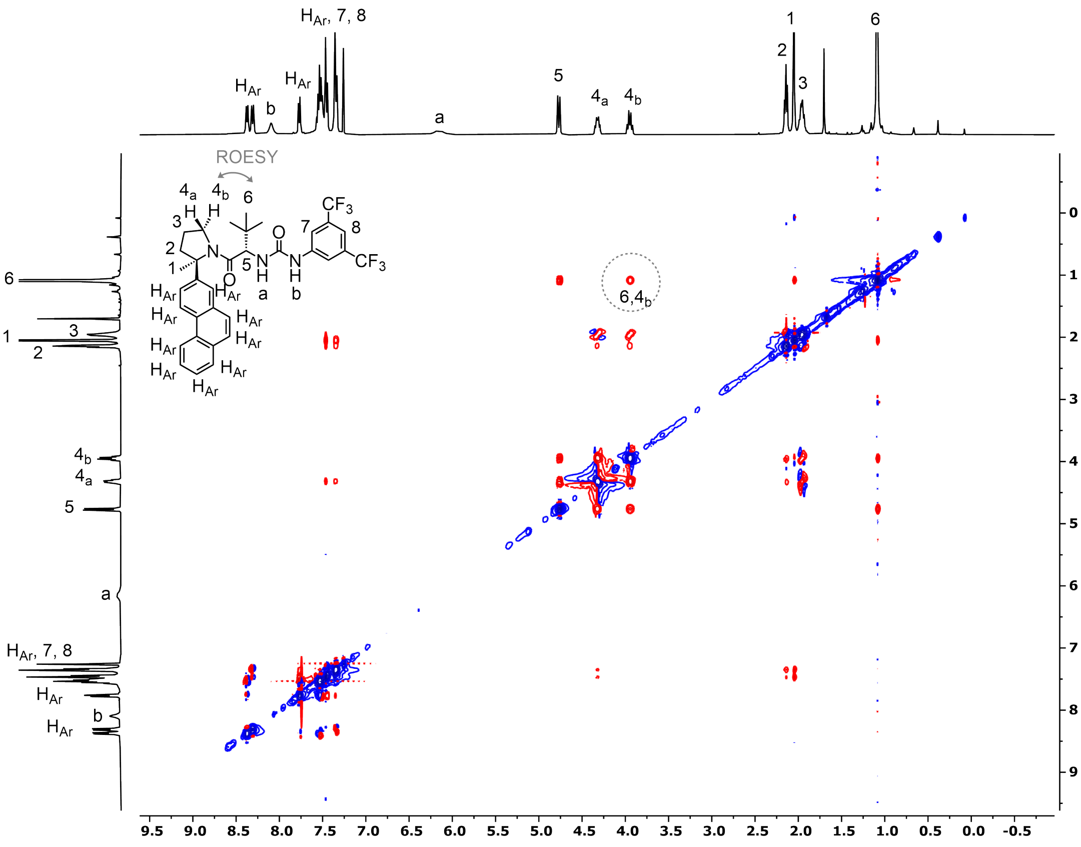

Figure S39. ROESY spectrum ($CDCl_3$) of Jacobsen catalyst **8.**

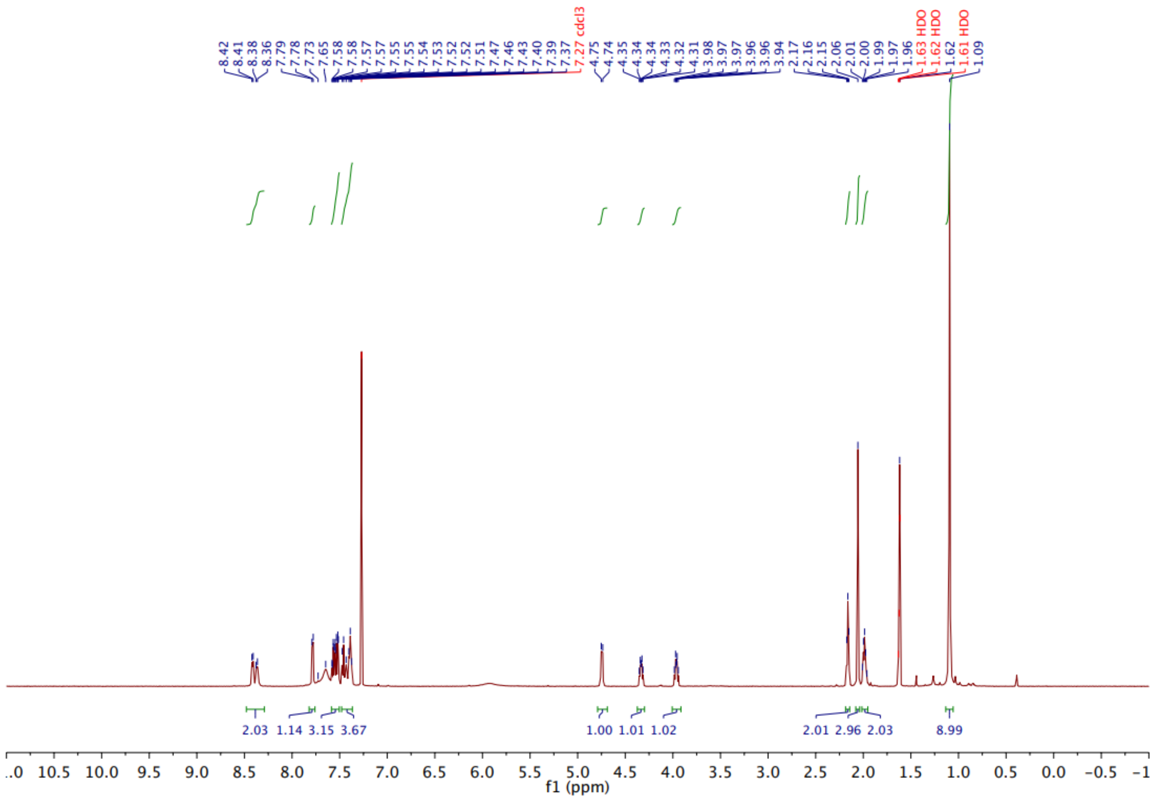

Figure S40. 400 MHz ^{1}H NMR spectrum ($CDCl_3$) of organocatalyst **8** reported in ref. [S2].
